# Multi-omics integration identifies serum FTL as a novel diagnostic biomarker for esophageal squamous cell carcinoma

**DOI:** 10.3389/fimmu.2026.1935836

**Published:** 2026-10-28

**Authors:** Wei-Zhong Chen, Shu-Lin Chen, Run-Kun Han, Xiao-Ru Wu, Jia-Cong Liang, Mian-Tao Wu, Guang-Yu Sun, Hao Chen

**Affiliations:** 1 Postdoctoral Research Station, Chaozhou People’s Hospital, Affiliated Chaozhou Hospital of Shantou University Medical College, Chaozhou, Guangdong, China; 2 Department of Clinical Laboratory, Affiliated Chaozhou Hospital of Shantou University Medical College, Chaozhou, Guangdong, China; 3 Department of Clinical Laboratory, State Key Laboratory of Oncology in South China, Guangdong Provincial Clinical Research Center for Cancer, Guangdong Esophageal Cancer Institute, Sun Yat-Sen University Cancer Center, Guangzhou, China; 4 School of Medical Technology, Guangdong Medical University, Dongguan, Guangdong, China; 5 Department of Gastroenterology, Chaozhou People’s Hospital, Affiliated Chaozhou Hospital of Shantou University Medical College, Chaozhou, Guangdong, China

**Keywords:** esophageal squamous cell carcinoma (ESCC), ferritin light chain (FTL), ferroptosis, proteomic, single-cell RNA sequencing (scRNA-seq)

## Abstract

**Background:**

Esophageal squamous cell carcinoma (ESCC) is a common malignant tumor in China, with a poor five-year survival rate due to the lack of effective early diagnostic methods. This study aimed to identify potential non-invasive biomarkers and clarify their underlying mechanisms.

**Methods:**

We employed an integrative approach combining serum proteomic profiling, bulk RNA sequencing, and single-cell RNA-seq (scRNA-seq) analyses. Quantitative proteomics was conducted on 15 serum samples of treatment-naive early-stage ESCC patients and 5 from healthy controls. Differentially expressed proteins (DEPs) were cross-referenced with differentially expressed genes (DEGs) from the GSE53624 dataset, and a diagnostic model was developed using LASSO regression. Cross-validation was performed using scRNA-seq data. The enzyme-linked immunosorbent assay (ELISA) was utilized to validate FTL in 155 serum samples from two centers. In silico FTL knockouts were conducted using scTenifoldKnk, followed by GO, KEGG, and PPI network analyses.

**Results:**

Proteomic profiling identified a total of 129 DEPs. Intersection with GSE53624 DEGs yielded 8 overlapping genes, and a 6-biomarker (TF, CHAD, CSRP1, FCGR3A, RCN1, FTL) diagnostic model was built, with AUC 0.882–1.0 across validation cohorts. scRNA-seq intersection analysis identified FTL as the only overlapping DEG, exhibiting high expression levels in ESCC epithelial cells and macrophages. FTL demonstrated strong standalone diagnostic performance, with AUC values ranging from 0.872 to 0.970 across three GEO datasets. ELISA validation yielded an AUC of 0.75 (95% CI: 0.674–0.816) and 0.838 (95% CI: 0.751–0.905) for early-stage ESCC, surpassing the performance of CEA and SCCA. The combination of FTL with SCCA further enhanced discriminatory efficacy. Mechanistically, FTL was associated with altered immune cell infiltration, characterized by increased resting dendritic cells and M2 macrophages and decreased follicular helper T cells, as well as activation of the ferroptosis pathway. In silico FTL knockouts revealed DEGs enriched in stress and metabolic biological processes, extracellular vesicles (cellular component), transcriptional regulation (molecular function, CHOP-ATF3 complex), IL-17/TNF pathways, and lipid/ROS-related pathways. Protein-protein interaction hubs included FOS, ATF3, NFKBIA, and CXCL8, with FTL linked to NFE2L2, TFRC, and TXNRD1.

**Conclusions:**

FTL represents a credible non-invasive serum diagnostic biomarker for ESCC. Computational analyses suggest it is a candidate molecule requiring further mechanistic and therapeutic investigation.

## Background

Esophageal squamous cell carcinoma (ESCC)is a prevalent malignant carcinoma in China (1, 2). Due to the lack of effective targeted approaches for early diagnosis and treatment, the five-year survival rate of ESCC patients remains dismal (3), emphasizing the urgent need to develop novel preventive and therapeutic strategies—an endeavor that underscores the critical importance of deepening our understanding of ESCC carcinogenesis (4).

Serum protein biomarkers exhibit excellent stability, ease of detection, and cost-effectiveness. Importantly, their non-invasive nature allows convenient longitudinal monitoring of therapeutic efficacy and patient prognosis. For these reasons, serum proteins represent one of the most ideal and widely applied classes of tumor biomarkers in clinical practice. In recent years, the rapid development of proteomic technologies has greatly facilitated the discovery of numerous potential protein biomarkers. Nevertheless, the validation of proteomic screening results still faces numerous challenges, particularly given the large number of candidate proteins. Therefore, the precise selection of dysregulated proteins for further experimental validation has become a critical step in biomarker research.

Multi-omics approaches have revolutionized the identification of clinically meaningful biomarkers and provided unprecedented insights into disease heterogeneity and progression. However, integrate proteomic and single-cell omic strategies for biomarker discovery remain scarce (5, 6). Proteomics provides a direct readout of functional molecules that drive phenotypic changes, which cannot be fully inferred from transcriptomic data alone. Due to post-transcriptional regulation, epigenetic modification, and post-translational modification, the abundance and activity of proteins often show limited consistency with mRNA levels. Therefore, proteomics serves as an indispensable complement to transcriptomics. The integration of proteomics and transcriptomics enables a more comprehensive and accurate dissection of molecular networks underlying biological processes, offering deeper insights into biomarker discovery and functional mechanism interpretation.

Our previous proteomic analyses comparing early-stage ESCC patients and healthy controls solely used simple differential analysis and candidate selection based on fold change and p-values; however, the large number of differentially expressed genes made individual validation impractical, with only FGL1 validated for its utility in ESCC screening (7). To address this limitation and meet the demand for better utilization of large-volume omics data while improving the precision and reliability of biomarker screening, this study integrated proteomics with bulk RNA sequencing and single-cell RNA sequencing (scRNA-seq) to systematically delineate the expression patterns of key candidate biomarkers.

This multi-omics integration facilitates the precise identification of biomarkers, enables cross-validation across omics platforms to reduce false-positive results and enhance robustness, and elucidates the molecular mechanisms underlying aberrant biomarker expression by profiling their distribution in specific cellular subpopulations of the tumor microenvironment, thereby prioritizing candidates with high clinical translational potential for early ESCC screening and diagnosis.

## Methods

### Proteomics and data analysis

Fifteen serum samples from treatment-naive patients with early-stage esophageal squamous cell carcinoma (ESCC) and five serum samples from healthy donors were collected, and quantitative proteomic assays were performed by Jingjie PTM BioLab Co., Ltd. (Hangzhou, China). Standard operating procedures were adopted for protein extraction, trypsin digestion, high-performance liquid chromatography (HPLC) fractionation, and liquid chromatography-tandem mass spectrometry (LC-MS/MS) analysis (8).

Differential expression analysis was conducted via Student’s t-test between the EC and healthy control groups, with proteins defined as differentially expressed when the *P*-value of the t-test was < 0.05 (7).

### Gene expression data collection and preprocessing

Gene expression data of ESCC were retrieved from The Cancer Genome Atlas (TCGA) database and the Gene Expression Omnibus (GEO) datasets GSE53624, GSE23400, and GSE20347. Differential gene expression analysis was performed between cancer samples (n=119) and normal samples (n=119) from the GSE53624 dataset via R packages: the limma package (version 3.52.1) was applied for microarray data processing, and the DESeq2 package (version 1.36.0) for the analysis of RNA-seq raw count data. Intergroup differential analysis (cancer vs. normal) was further conducted using the limma package, with the screening criteria set as |log2FC| > 2 and p-value < 0.05 (9).

### Construction of the predictive model

Differentially expressed proteins (DEPs) derived from serum proteomic analysis between early ESCC and HC groups were stratified into upregulated and downregulated subsets, and then intersected with the differentially expressed genes (DEGs) identified from bulk RNA-seq analysis between cancer and normal groups. Ultimately, 5 upregulated genes and 3 downregulated genes were obtained. Samples from the GSE53624 dataset were randomly divided into two subgroups at a ratio of 7:3, with one serving as the training set and the other as the internal validation set. All samples from GSE23400, GSE20347 and TCGA-ESCA datasets were used as independent external validation sets, respectively. A diagnostic model was constructed using the multivariate generalized linear model based on LASSO regularization (GLMNET) with the model type set as binomial, where the diagnostic status of ESCC was designated as the response variable. The optimal penalty parameter λ was selected via cross-validation, and the model performance was validated on both the training set and the internal validation set. The performance of the constructed model was further evaluated using the receiver operating characteristic (ROC) curve and the area under the curve (AUC) value.

### Single-cell data preprocessing

GSE196756 scRNA-seq data (6 samples: 3 ESCC, 3 normal) were downloaded from GEO; after QC (filtering non-expressing barcodes via DropletUtils (10), UMI-based cell exclusion, and gene expression quantification/summary with scater (11), expression matrices were normalized using Seurat’s NormalizeData (12), followed by identification of top 2000 variable genes via FindVariableFeatures, data scaling with ScaleData, and linear dimensionality reduction by RunPCA (Seurat); high-variance PCs were used for cell clustering through Seurat’s FindNeighbors and FindClusters, with UMAP visualization via RunUMAP; cluster-specific marker genes (top 500 logFC-ranked DEGs) were identified using Seurat’s FindMarkers (log2FC ≥ 0.25, min cluster expression proportion ≥ 0.1, p ≤ 0.05) for cell annotation; finally, epithelial cells were subjected to ESCC vs. normal differential analysis (|log2FC| >log2(1.2), p < 0.05).

### Laboratory analysis

Patients with histopathologically confirmed ESCC(n=78)were enrolled, with no prior antitumor therapy and no other concurrent malignancies. Healthy controls (n = 77) were free of malignant diseases, autoimmune disorders, severe infections, and acute inflammatory conditions, with normal physical examination and routine laboratory results. [From two centers: Sun Yat-sen University Cancer Center (SYSUCC) and Chaozhou People’s Hospital (CZPH)] All enrolled ESCC patients were clinically and pathologically staged according to the 8th edition American Joint Committee on Cancer (AJCC) TNM staging system for esophageal cancer. In this study, stage I–II disease was uniformly defined as early-stage ESCC.

Approximately 4 mL of fasting peripheral venous blood was collected and immediately centrifuged at 3500 rpm for 10 min at 20–22 °C. Serum was separated and subsequently transferred to −80 °C for long-term storage. Serum concentrations of CEA and SCC-Ag were measured by electrochemiluminescence immunoassay using a Roche cobas e 801 analyzer (Roche Diagnostics, Mannheim, Germany). Serum FTL concentration was measured by Human FTL ELISA Kit (Cat. No. CSB-EL009049HU-IS, Cusabio Biotech Co., Ltd., Wuhan, China). The assay was strictly performed in accordance with the manufacturer’s standard operating protocols (https://www.cusabio.com/). Prior to detection, all serum samples were pre-diluted at a ratio of 1:500.

### Immune infiltration analysis

The CIBERSORT algorithm was employed to evaluate the infiltration levels of 22 immune cell subsets in each ESCC sample from the GSE53624 dataset. Samples were divided into high-expression and low-expression groups based on the median expression level of FTL. The differences in immune infiltration levels between the two groups were compared using the Wilcoxon rank-sum test. We also performed Pearson correlation analyses to evaluate the associations between continuous FTL expression levels and the CIBERSORT-derived relative fractions of the 22 immune cell subsets.

### Ferroptosis analysis

The ferroptosis pathway activity score was calculated based on the expression profiles of ferroptosis driver genes and suppressor genes (13). Two independent gene sets were extracted from bulk RNA-seq data and single-cell RNA-seq data, respectively. The pathway activity score for each sample was defined as the average value of the expression levels of genes within the corresponding gene set. Similarly, the Wilcoxon test was used to compare the differences in pathway activity scores between the high and low FTL expression groups.

### Virtual knockout of FTL using scTenifoldKnk

scTenifoldKnk was used for in silico FTL knockout (14); briefly, a wild-type gene regulatory network (GRN) was constructed from preprocessed GSE196756 data, followed by five independent virtual FTL knockouts (setting FTL’s GRN adjacency matrix row to zero) to ensure reproducibility. Differentially regulated genes (DRGs) were identified with |Z| > 2 and perturbation percentage > 10% across replicates, and merged DRGs were used for subsequent analyses. Gene Ontology (GO) and KEGG pathway analyses were performed with FDR < 0.05 to explore DEG-related functions and pathways, while the STRING database (confidence score ≥ 0.7) was employed to construct protein-protein interaction (PPI) networks, which were visualized and analyzed via Cytoscape to identify key modules and hub genes.

### Statistical analysis

All statistical analyses were performed using R software (version 4.2.0). Differential expression analysis between ESCC and healthy control groups in proteomic data was conducted using Student’s t-test, with proteins considered differentially expressed when P < 0.05. For bulk RNA-seq data, differential expression analysis was performed using the limma package (version 3.60.6) for microarray data and the DESeq2 package (version 1.36.0) for RNA-seq raw count data, with screening criteria set as |log2FC| > 2 and P value < 0.05. For single-cell RNA-seq data, differential expression analysis between ESCC and normal epithelial cells was performed using the FindMarkers function in Seurat, with |log2FC| > log2(1.2) and P value < 0.05.

The diagnostic model was constructed using LASSO logistic regression via the glmnet package, with the optimal penalty parameter λ selected by 10-fold cross-validation. Model performance was evaluated using ROC curves and AUC values. ROC curve analyses for serum CEA, SCCA, FTL and the combined Panel 2 biomarker were performed using MedCalc software. The area under the curve (AUC), the optimal cutoff value (determined by maximizing Youden’s index), and the corresponding sensitivity and specificity were all directly output by MedCalc based on the ROC analysis. Panel 2 represents the predicted probability derived from logistic regression combining serum FTL and SCCA.

Immune infiltration levels were compared between high- and low-FTL expression groups using the Wilcoxon rank-sum test. Ferroptosis pathway activity scores were compared similarly. All tests were two-sided, and a P-value < 0.05 was considered statistically significant.

## Results

### Integrated analysis of serum proteome and bulk RNA-seq

Fifteen serum samples from patients with early-stage ESCC and five serum samples from healthy donors were collected at SYSUCC for quantitative proteomic analysis. (**Figure 1**) A total of 1243 proteins were quantified, and after excluding the data with missing quantitative information across samples, 710 serum proteins with complete quantitation were finally identified. Student’s t-test comparing the early-stage ESCC group and HC group showed 129 proteins (DEPs) with significant expression differences (P<0.05), including 71 upregulated and 58 downregulated proteins in early-stage ESCC patients (**Figure 2**).

**Figure 1:**
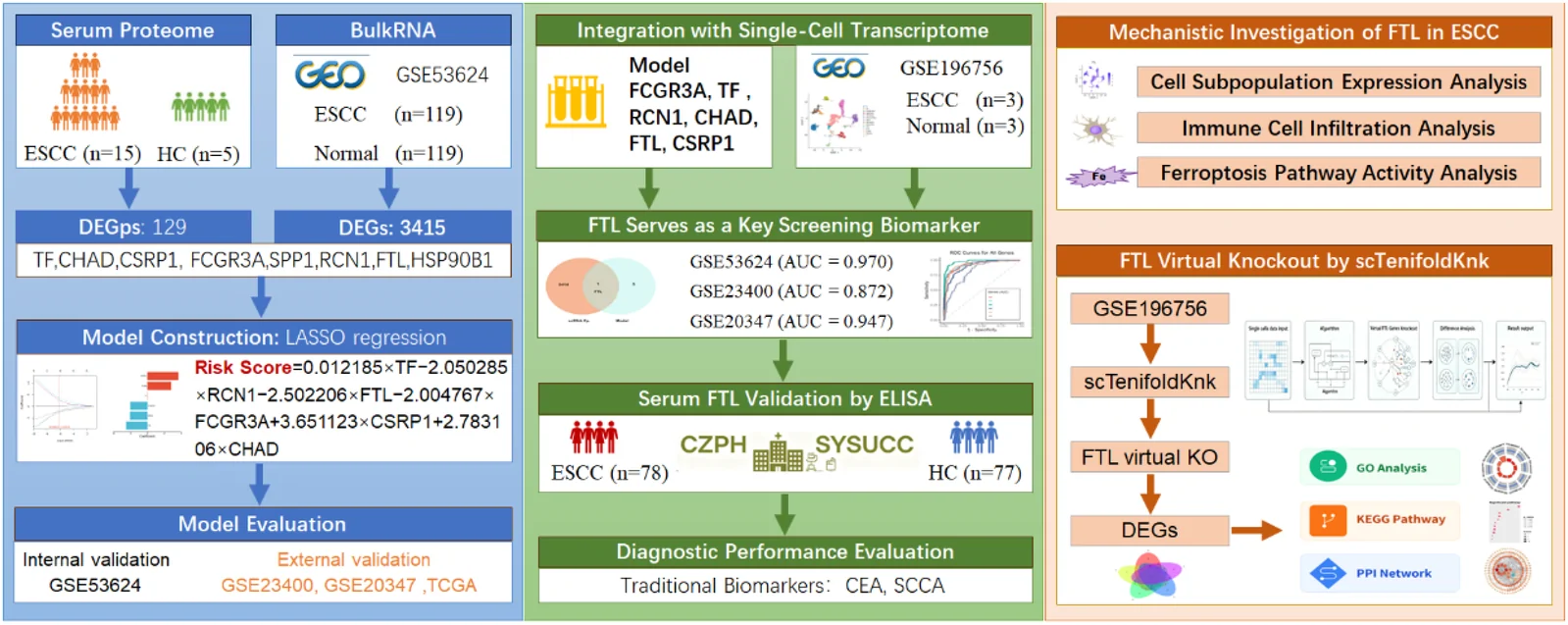
Overview of the workflow.

**Figure 2:**
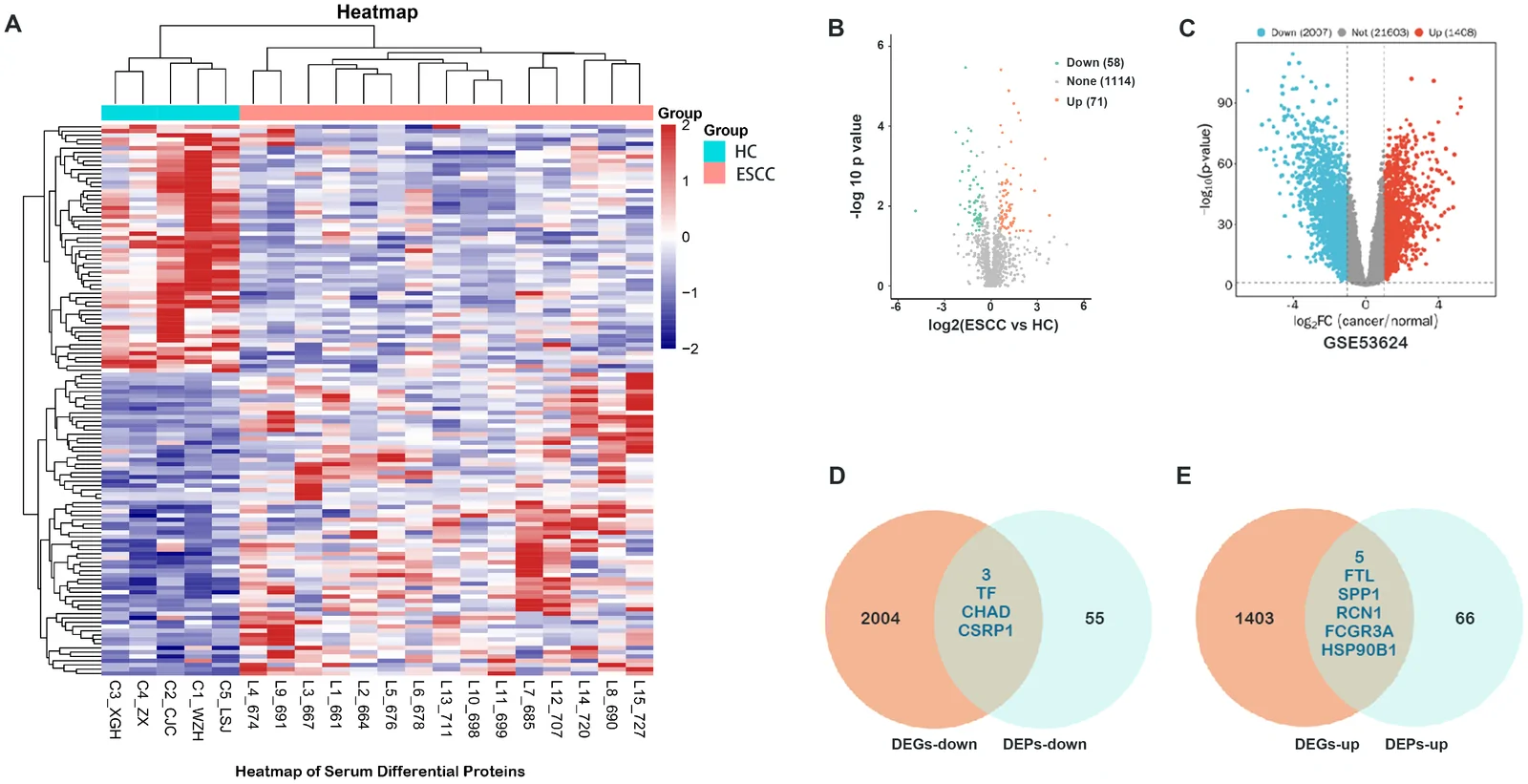
Integrated analysis of plasma proteomic and bulk RNA profiles in ESCC. **(A)** Heatmap of differentially expressed plasma proteins (DEPs). **(B)** Volcano plot of DEPs. **(C)** Volcano plot of differentially expressed genes(DEGs) from bulk RNA-seq. **(D)** Venn diagram of downregulated proteins and genes. **(E)** Venn diagram of upregulated proteins and genes.

A total of 3415 DEGs from GSE53624 dataset, with 1408 upregulated and 2007 downregulated, were intersected with the DEPs from the serum proteome, yielding 5 upregulated genes and 3 downregulated genes in the overlapping set (**Figure 2**).

### Model construction and evaluation

Samples from the GSE53624 dataset were randomly split into two subsets at a 7:3 ratio. Six biomarkers (TF, CHAD, CSRP1, FCGR3A, RCN1, FTL) were screened out via LASSO regression, and a tissue-based transcriptomic diagnostic model (guided by serum proteomic candidates) was subsequently constructed based on these biomarkers (Risk Score=0.012185×TF−2.050285×RCN1−2.502206×FTL−2.004767×FCGR3A+3.651123×CSRP1 + 2.783106×CHAD). (**Figures 3A, B**) The model achieved an AUC of 1.0 in the training subset and 0.992 in the internal validation subset of GSE53624. (**Figure 3C**) For independent external validation, AUCs were 0.972 in GSE23400, 0.934 in GSE20347, and 0.882 in TCGA-ESCC (**Figures 3D–F**).

**Figure 3:**
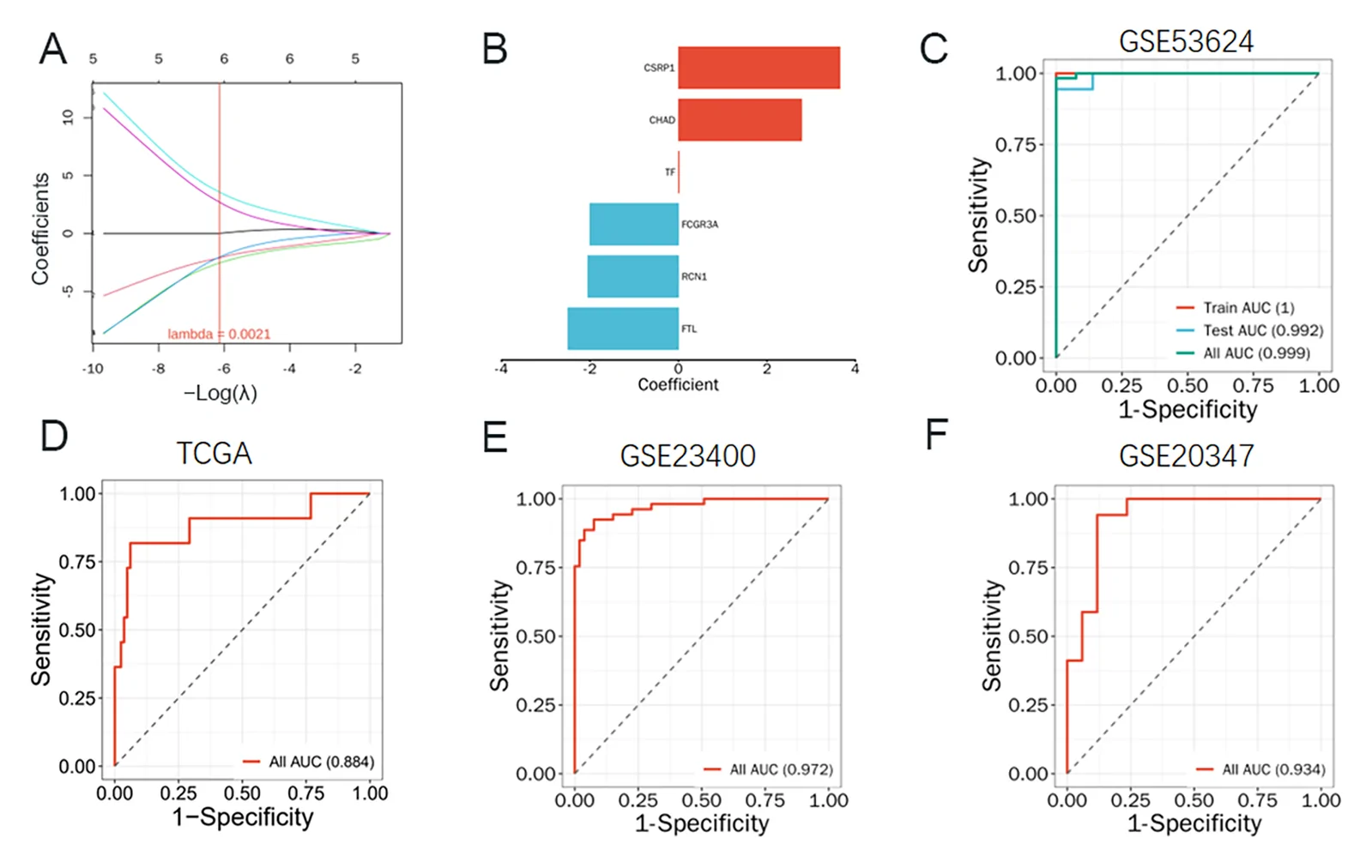
Construction and evaluation of the six-biomarker diagnostic model. **(A)** Stepwise LASSO regression plot for screening candidate biomarkers. **(B)** Coefficient plot of the six selected biomarkers (TF, CHAD, CSRP1, FCGR3A, RCN1, FTL). **(C)** ROC curve of the diagnostic model in the training and internal validation subset of GSE53624. **(D)** ROC curve of the diagnostic model in the independent external validation dataset TCGA-ESCC. **(E)** ROC curve of the diagnostic model in the independent external validation datasets GSE23400. **(F)** ROC curve of the diagnostic model in the independent external validation datasets GSE20347.

### Intersection analysis of the serum protein model and single-cell transcriptome

To screen for candidate molecules with consistent associations in both the serum protein model and single-cell sequencing data (**Figures 4A, B**), we performed an intersection analysis between the 6 biomarkers of the established model and the differentially expressed gene (scRNA Ep) data from ESCC single-cell transcriptomes for esophageal cancer and normal epithelial cells (**Figure 4C**). A single differentially expressed gene, FTL, was identified from the intersection. (**Figure 4D**) This gene was highly expressed in epithelial cells and macrophages, with a significant expression difference between normal and tumor epithelial cells (**Figures 4E, F**).

**Figure 4:**
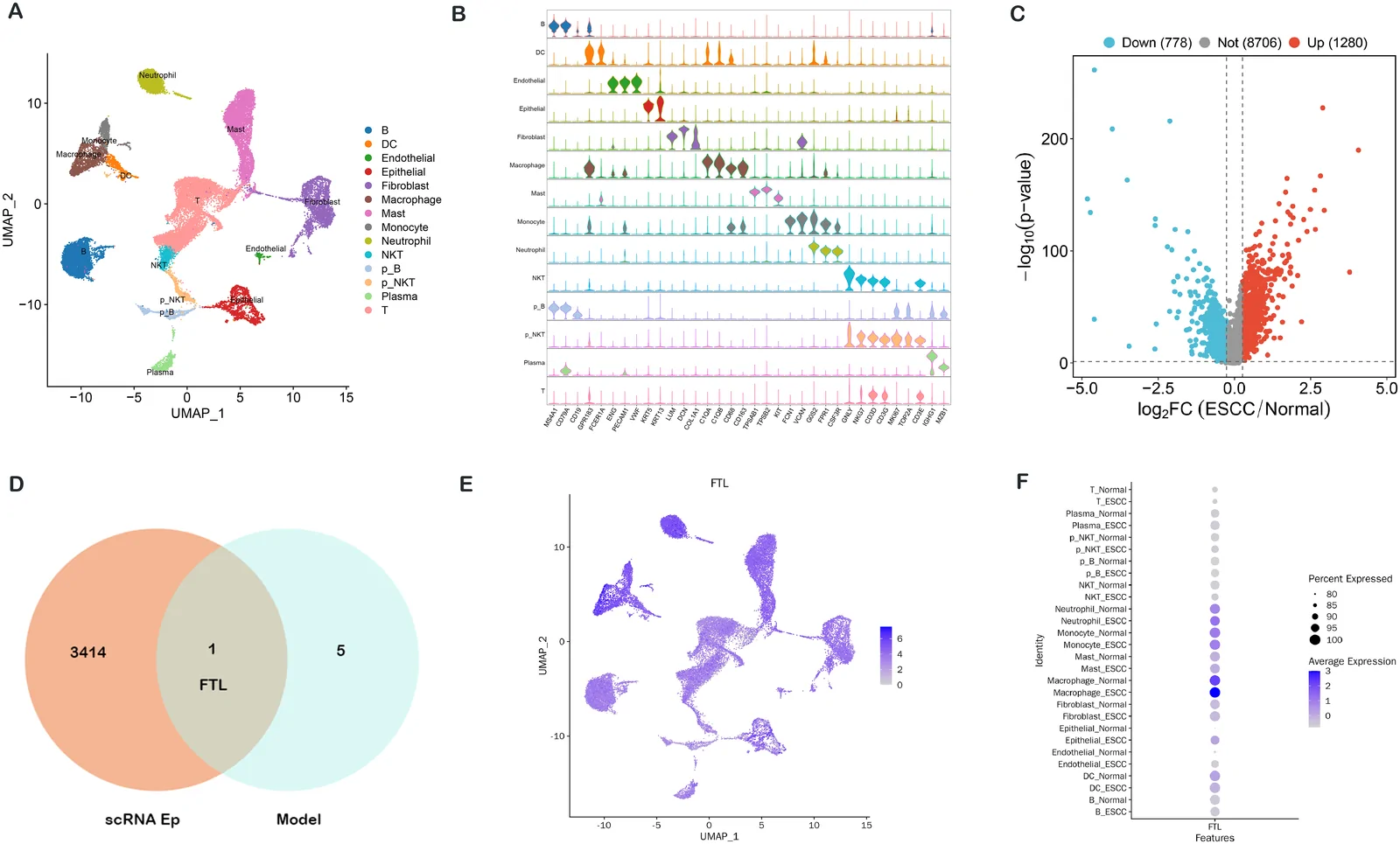
Integrated single-cell transcriptomic analysis identifies FTL as a critical gene in ESCC. **(A)** UMAP of cell populations in ESCC scRNA-seq data. **(B)** Cell-type specific expression of marker genes. **(C)** Volcano plot of differentially expressed gene (scRNA Ep) from ESCC single-cell transcriptomes for ESCC and normal epithelial cells. **(D)** Venn diagram overlapping scRNA-seq epithelial genes with model candidates, pinpointing FTL as a shared hit. **(E)** UMAP of FTL expression at single-cell level. **(F)** Dot plot of FTL expression across cell types and conditions.

### FTL as a potential biomarker for ESCC

We assessed the standalone diagnostic performance of FTL at the transcript level in GEO datasets, which exhibited favorable efficacy in GSE53624 (AUC = 0.970, 95%CI: 0.944-0.986), GSE23400 (AUC = 0.872, 95%CI: 0.799-0.932), and GSE20347 (AUC = 0.947, 95%CI: 0.805-0.973), accompanied by corresponding sensitivity, specificity, PPV, NPV, PLR, and NLR values (**Supplementary Table S1**; **Figures 5A–C**).

**Figure 5:**
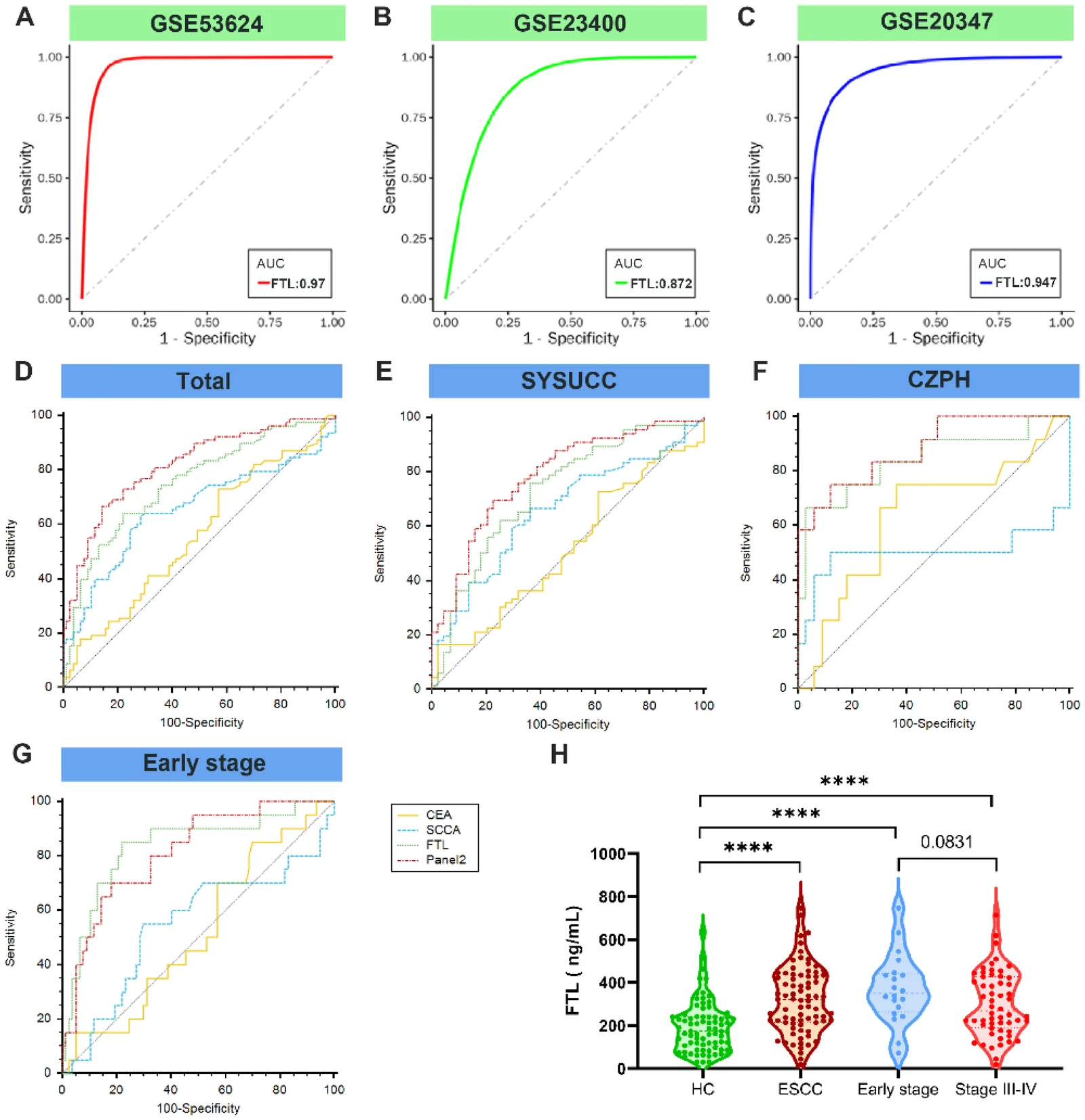
Diagnostic performance of FTL in ESCC. **(A-C)** Receiver operating characteristic (ROC) curves and corresponding area under the curve (AUC) values of FTL for ESCC diagnosis in public datasets. **(D-G)** ROC curves of FTL, conventional tumor markers including CEA, SCCA and Panel 2 for ESCC diagnosis. **(D)** Combined cohort from SYSUCC and CZPH; **(E)** Cohort from SYSUCC alone; **(F)** Cohort from CZPH alone; **(G)** ROC curve of FTL in the early-stage ESCC subgroup. **(H)** Violin plot showing the differential expression distribution of FTL between HC and patients with ESCC. Panel 2: FTL combined with SCCA. ***, p < 0.001.

Subsequently, we further evaluated the diagnostic performance of serum FTL concentration as a screening marker for ESCC, using serum samples from two independent centers (CZPH and SYSUCC), and its performance was compared with that of conventional ESCC biomarkers (**Table 1**; **Supplementary Table S2**). Results showed that serum FTL concentration was significantly higher in ESCC patients than in normal controls (P < 0.001), with an AUC of 0.75 (95% CI: 0.674~0.816) for the overall population and 0.838 (95% CI: 0.751~0.905) for early-stage ESCC, which was superior to conventional serum biomarkers (CEA, SCCA). (**Figures 5D–H**) Additionally, among all patients, the combined diagnostic Panel 2 (FTL + SCCA) demonstrated significantly superior diagnostic performance relative to FTL alone and all other single conventional biomarkers. However, in early-stage ESCC, the AUC values of Panel 2 and FTL alone were comparable (0.824 vs. 0.838), with no statistically significant difference observed between the two AUCs (p = 0.6694). Raw sample-level counts are provided in **Supplementary Table S3**. Importantly, both Panel 2 and FTL alone outperformed conventional biomarkers in both all patients and early-stage ESCC subgroups (**Table 2**).

**Table 1:** Baseline characteristics of the two centers.

Variable	Total				HC				ESCC			
	Overall	HC	ESCC	p-value	Overall	CZPH	SYSUCC	p-value	Overall	CZPH	SYSUCC	p-value
	n = 155	n = 77 (50%)	n = 78 (50%)		n= 77	n = 33 (43%)	n = 44 (57%)		n = 78	n = 12 (15%)	n = 66 (85%)	
Sex				>0.999				0.939				>0.999
Female	38 (24.52%)	19 (24.68%)	19 (24.36%)		19 (24.68%)	8 (24.24%)	11 (25.00%)		19 (24.36%)	3 (25.00%)	16 (24.24%)	
Male	117 (75.48%)	58 (75.32%)	59 (75.64%)		58 (75.32%)	25 (75.76%)	33 (75.00%)		59 (75.64%)	9 (75.00%)	50 (75.76%)	
Age	62.29 (9.07)	61.82 (8.49)	62.76 (9.64)	0.521	61.82 (8.49)	60.85 (6.80)	62.55 (9.58)	0.389	62.76 (9.64)	69.42 (7.57)	61.55 (9.52)	0.008
FTL (ng/mL)	230	173.64	320.29	<0.001	173.64	203.63	162.78	0.817	320.29	380.77	302.00	0.166
	[138.51, 348.25]	[106.57, 240.18]	[215.79, 432.94]		[106.57, 240.18]	[118.40, 239.51]	[95.09, 243.51]		[213.78, 434.32]	[236.81, 501.54]	[207.91, 430.30]	
CEA (ng/mL)	2.41	2.21	2.43	0.226	2.21	2.03	2.38	0.431	2.43	3.52	2.37	0.289
	[1.44, 3.87]	[1.35, 3.70]	[1.63, 3.94]		[1.35, 3.70]	[1.30, 3.70]	[1.59, 3.71]		[1.63, 3.94]	[2.22, 5.53]	[1.63, 3.76]	
SCCA (ng/mL)	1.38	1.17	1.67	<0.001	1.17	1.15	1.20	0.23	1.67	1.42	1.67	0.25
	[0.98, 2.13]	[0.93, 1.60]	[1.11, 3.09]		[0.93, 1.60]	[0.87, 1.38]	[0.99, 1.71]		[1.11, 3.09]	[0.54, 3.20]	[1.17, 3.09]	
Panel2	0.45	0.31	0.7	<0.001	0.31	0.33	0.31	0.866	0.7	0.8	0.69	0.633
	[0.30, 0.75]	[0.19, 0.47]	[0.43, 0.86]		[0.19, 0.47]	[0.21, 0.40]	[0.19, 0.50]		[0.43, 0.86]	[0.46, 0.88]	[0.43, 0.85]	

ESCC, Esophageal squamous cell carcinoma; HC, healthy controls; SYSUCC, Sun Yat-sen University Cancer Center; CZPH, Chaozhou People’s Hospital; n (%) or Median [IQR].

**Table 2:** Diagnostic performance of serum FTL and conventional tumor markers in ESCC.

Biomarker	AUC (95%CI)		Criterion	Sensitivity	95% CI	Specificity	95% CI	+LR	95% CI	-LR	95% CI
Total											
CEA	0.556	0.474 - 0.636	>1.8 *	73.08	61.8 - 82.5	42.86	31.6 - 54.6	1.28	1.0 - 1.7	0.63	0.4 - 1.0
			>5.0	19.23	11.2 - 29.7	88.31	79.0 - 94.5	1.65	1.0 - 2.6	0.91	0.5 - 1.7
SCCA	0.654	0.573 - 0.728	>1.45 *	62.82	51.1 - 73.5	71.43	60.0 - 81.2	2.2	1.8 - 2.7	0.52	0.3 - 0.8
FTL	0.75	0.674 - 0.816	>241.079 *	64.1	52.4 - 74.7	77.92	67.0 - 86.6	2.9	2.4 - 3.6	0.46	0.3 - 0.8
Panel 2	0.816	0.746 - 0.874	>0.5447 *	66.67	55.1 - 76.9	85.71	75.9 - 92.6	4.67	3.9 - 5.6	0.39	0.2 - 0.7
Early stage											
CEA	0.522	0.419 - 0.623	>1.4 *	86.36	65.1 - 97.1	29.87	20.0 - 41.4	1.23	0.8 - 1.8	0.46	0.2 - 1.3
			>5.0	13.64	2.9 - 34.9	88.31	79.0 - 94.5	1.17	0.4 - 3.3	0.98	0.5 - 1.8
SCCA	0.584	0.481 - 0.682	>1.4 *	59.09	36.4 - 79.3	70.13	58.6 - 80.0	1.98	1.4 - 2.9	0.58	0.3 - 1.1
FTL	0.838	0.751 - 0.905	>241.079 *	86.36	65.1 - 97.1	77.92	67.0 - 86.6	3.91	3.2 - 4.8	0.17	0.06 - 0.5
Panel 2	0.824	0.735 - 0.893	>0.5229 *	72.73	49.8 - 89.3	81.82	71.4 - 89.7	4	3.0 - 5.3	0.33	0.1 - 0.8

The serum concentrations of CEA, SCCA, and FTL are presented in ng/mL. *Optimal cutoff determined by maximizing Youden’s index in MedCalc (interpolated threshold). Panel 2: diagnostic model combining SCCA and FTL.

### FTL expression correlates with immune cell infiltration in ESCC

To explore the association between FTL expression and immune cell infiltration in ESCC, samples from GSE53624 dataset were stratified into FTL high- and low-expression groups based on the median FTL level. High FTL expression was associated with increased infiltration of resting dendritic cells and M2-type macrophages, and reduced follicular helper T (Tfh) cell infiltration (**Figures 6A–D**). To further validate these associations using continuous FTL expression, Pearson correlation analyses were performed in the GSE53624 dataset to relate FTL mRNA expression to CIBERSORT-estimated relative fractions of immune cell subsets. Consistent with the subgroup analysis, FTL expression was negatively correlated with Tfh cells (R = −0.348, p < 0.001) and positively correlated with resting dendritic cells (R = 0.281, p = 0.002), while no significant correlation was found with M2 macrophages (R = 0.145, p = 0.116) (**Figures 6E–G**).

**Figure 6:**
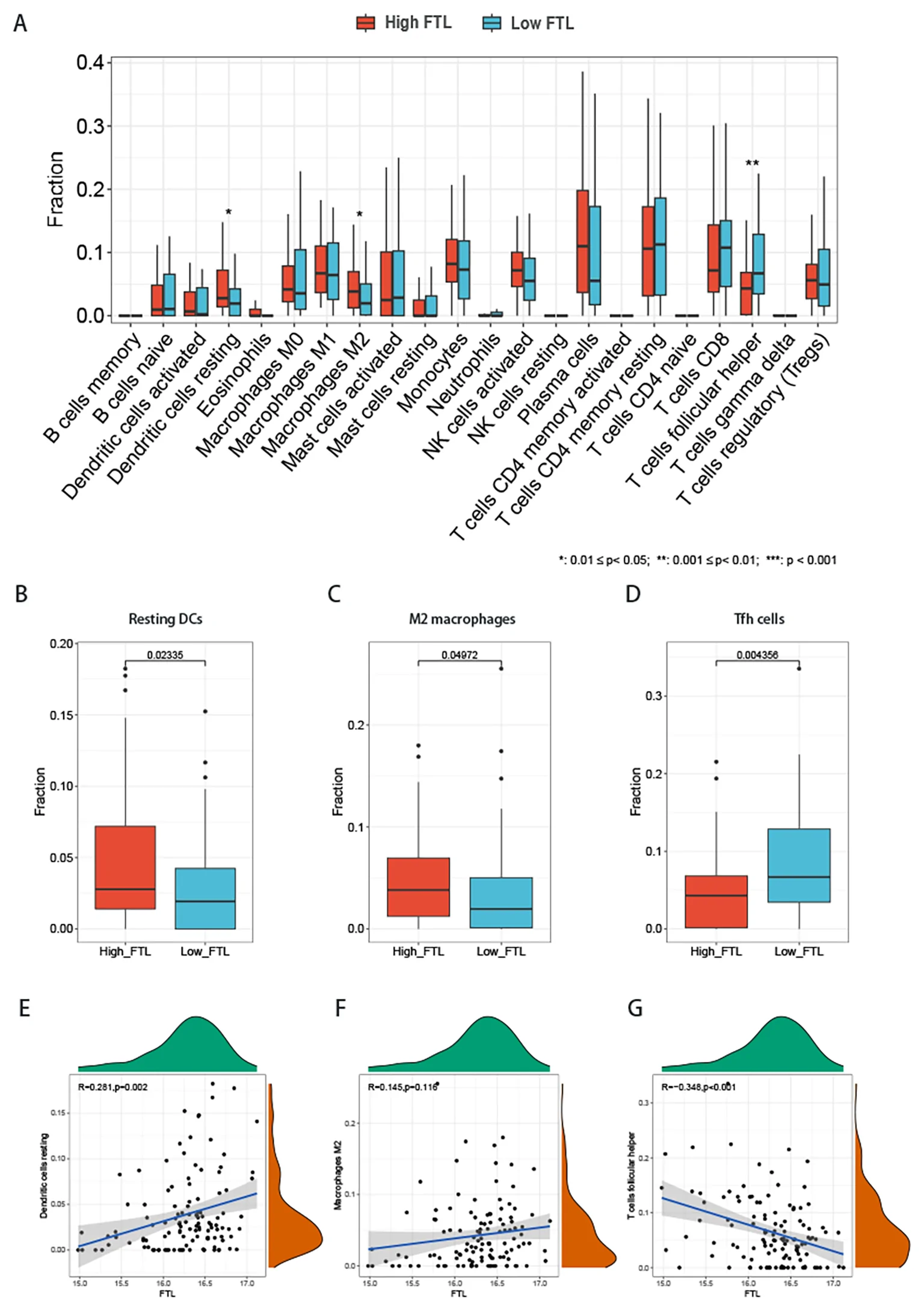
The differences of immune cell infiltration between the high and low FTL samples. **(A)** Box plots comparing the relative fractions of 22 tumor-infiltrating immune cell subsets between patients with high FTL expression (High-FTL, red) and low FTL expression (Low-FTL, blue) in the ESCC cohort. **(B-D)** Detailed box plots showing the significantly different fractions of resting dendritic cells (Resting DCs), M2 macrophages, and follicular helper T cells (Tfh cells) between the High-FTL and Low-FTL groups. **(E-G)** Scatter plots showing Pearson correlations between continuous FTL expression level and relative fractions of M2 macrophages, follicular helper T cells, and resting dendritic cells in ESCC samples. All immune-cell-related analyses were performed using the GSE53624 ESCC cohort. R: Pearson correlation coefficient. Data are presented as median with interquartile range (IQR), and whiskers extend to 1.5×IQR. *P < 0.05, **P < 0.01, as determined by the Wilcoxon rank-sum test.

### FTL expression is associated with ferroptosis pathway activity in ESCC

Subsequently, we further explored the association between high and low FTL expression groups and the activity score of the ferroptosis pathway in ESCC, which was evaluated based on the expression profiles of ferroptosis driver genes and suppressor genes. Specifically, we analyzed two independent gene sets derived from bulk RNA sequencing data and single-cell RNA sequencing (scRNA-seq) data, respectively. (**Figure 7**) The results showed that in both gene sets, the high FTL expression group exhibited significantly higher expression levels of both ferroptosis driver genes and suppressor genes compared with the low FTL expression group, and the differences between the two groups were statistically significant (all P < 0.05). Notably, the pathway activity score of ferroptosis suppressor genes in the high FTL expression group was significantly higher than that of ferroptosis driver genes, suggesting that FTL may be involved in the regulation of ferroptosis in ESCC by predominantly upregulating the expression of ferroptosis suppressor genes.

**Figure 7:**
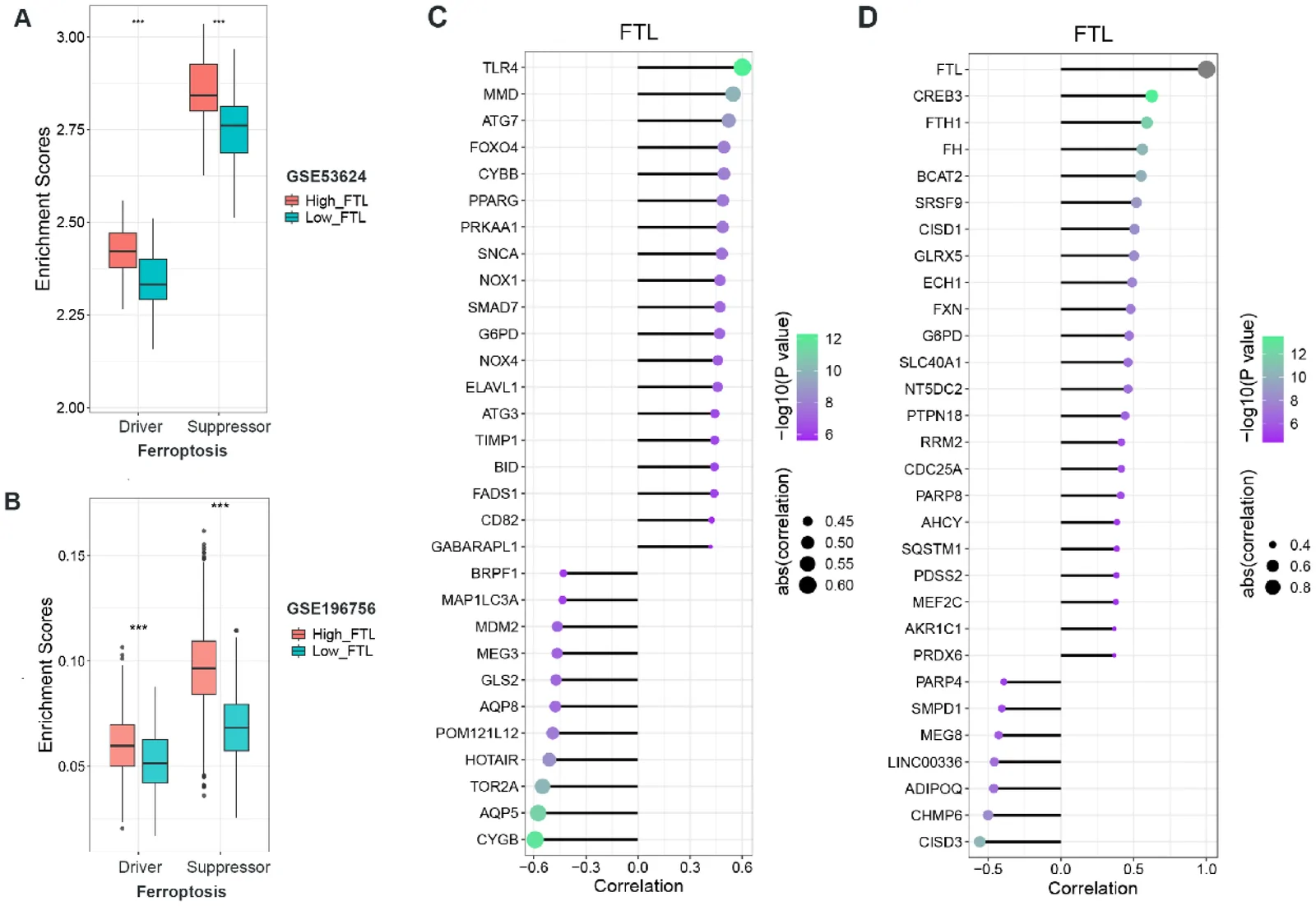
FTL correlates with ferroptosis gene expression in ESCC. **(A)** Box plots comparing single-sample gene set enrichment analysis (ssGSEA) scores of ferroptosis driver and suppressor gene sets between patients with high (red) and low (teal) FTL expression in the GSE53624 bulk ESCC cohort. **(B)** Corresponding box plots of AUCell enrichment scores for ferroptosis driver and suppressor gene sets in high vs low FTL groups from the GSE196756 single-cell RNA-seq ESCC dataset. **(C, D)** Lollipop plot illustrating the correlation between FTL expression and the expression levels of ferroptosis driver genes **(C)** and suppressor genes **(D)** in ESCC. ***, p < 0.001.

### Identification of differentially regulated genes and functional characteristics following FTL virtual knockout

In silico FTL knockout was performed in five replicates using scTenifoldKnk. No consistent DEGs were found across all runs; thus, the union of DEGs was analyzed. (**Figure 8A**) GO enrichment showed top BP terms related to stress and metabolic responses, with enrichment in extracellular vesicles (CC) and transcriptional regulation (MF), most notably the CHOP-ATF3 complex. (**Figures 8B, C**) KEGG analysis revealed enrichment in inflammatory/immune pathways (IL-17, TNF, NF-κB, apoptosis), with IL-17 and TNF signaling as the most prominent. Lipid/atherosclerosis and ROS-related pathways were also enriched, indicating oxidative stress and metabolic dysregulation (**Figures 8D, E**). PPI network analysis identified FOS, ATF3, NFKBIA, and CXCL8 as high-degree hubs. *NFE2L2*, *TFRC*, and *TXNRD1* were closely connected to FTL, with *TFRC* and *TXNRD1* linking the network to iron metabolism and oxidative stress (**Figure 8F**). Collectively, these computational analyses suggest that FTL loss may be associated with transcriptomic rewiring in ESCC, potentially mediated through inflammatory, immune, and oxidative stress pathways.

**Figure 8:**
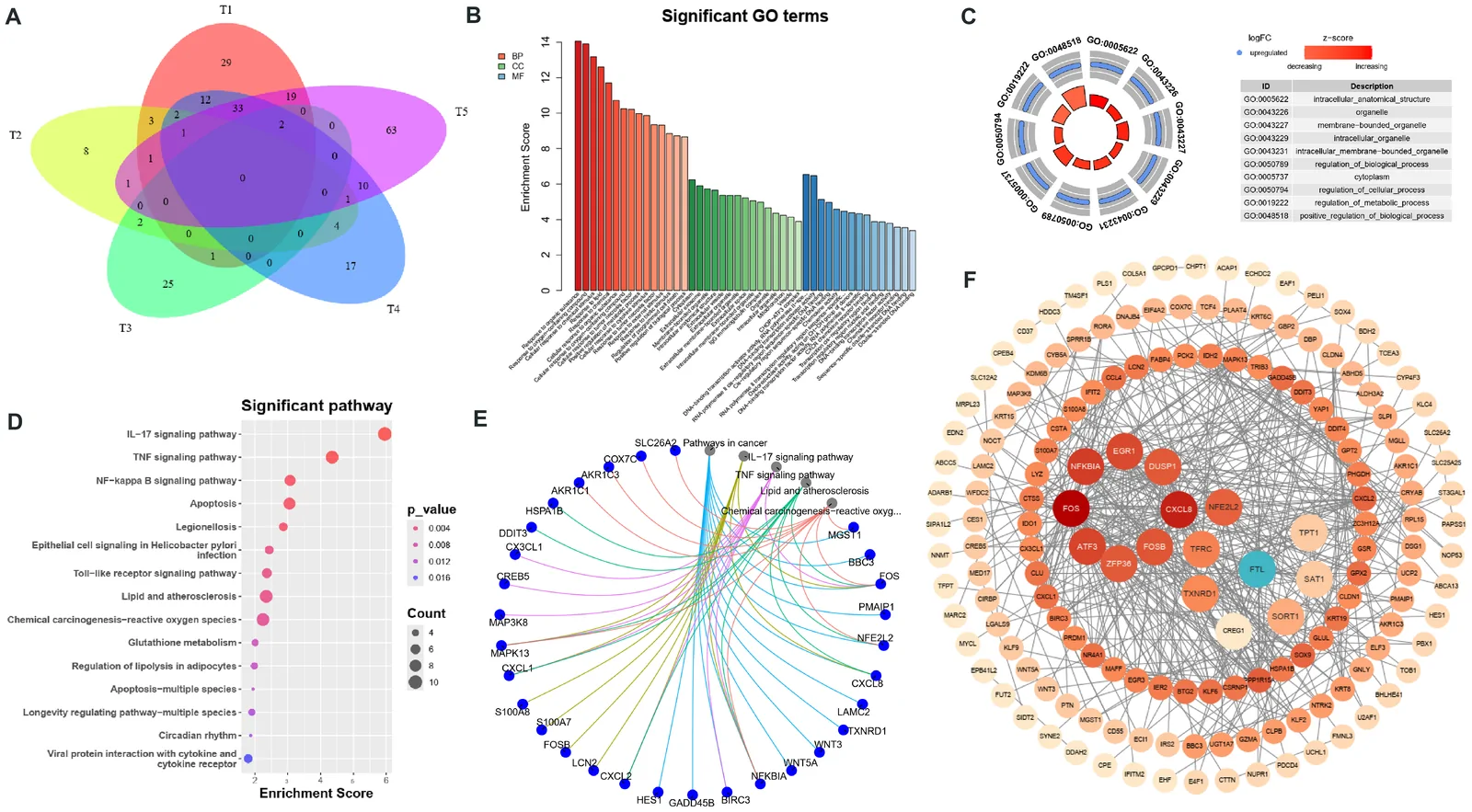
FTL virtual knockout and functional analysis results. **(A)** Venn diagram showing the overlap of differentially regulated genes (DRGs) identified from five independent in silico FTL knockout replicates using scTenifoldKnk in the GSE196756 single-cell dataset. **(B, C)** Gene Ontology (GO) enrichment analysis of merged DRGs after five FTL virtual knockouts. **(D, E)** Kyoto Encyclopedia of Genes and Genomes (KEGG) pathway enrichment analysis of merged DRGs. **(F)** Protein-protein interaction (PPI) network of merged DRGs constructed using the STRING database and visualized by Cytoscape. The size of the circles in **(F)** is not meaningful and only serves to highlight genes with obvious Degree values; the color of the circles gradually deepens with the increase of Degree. The FTL gene is marked in cyan-blue.

## Discussion

The prognosis of ESCC is adversely affected by the absence of reliable early screening biomarkers and an incomplete understanding of the molecular mechanisms underlying its progression, emphasizing the critical need for further research in this area (14, 15). In this study, we combined serum proteomic profiling, bulk RNA sequencing, and single-cell transcriptome analysis to find early-stage ESCC diagnostic biomarkers and explore their functions. We identified 129 differentially expressed serum proteins (71 upregulated, 58 downregulated) between early-stage ESCC patients and healthy controls. By intersecting these with DEGs from the GSE53624 dataset, we found eight overlapping genes. Using LASSO regression, we developed a diagnostic model with six biomarkers (TF, CHAD, CSRP1, FCGR3A, RCN1, FTL), showing excellent diagnostic performance in validation cohorts. Intersection analysis with single-cell data highlighted FTL as a key gene, suggesting its crucial role in ESCC pathogenesis. FTL, as a key subunit of the iron storage protein complex, plays a central role in maintaining iron homeostasis and antioxidant defense (16). Numerous studies have shown that FTL is aberrantly expressed in various malignant tumors and participates in tumor initiation and progression through mechanisms such as regulating cell cycle, ferroptosis, epithelial-mesenchymal transition, and the immune microenvironment (17–19).

Concurrently, we further validated the independent diagnostic performance of FTL, with our findings demonstrating its diagnostic efficacy across three independent datasets: GSE53624 (AUC = 0.970), GSE23400 (AUC = 0.872), and GSE20347 (AUC = 0.947). High AUC values observed in GEO datasets reflect FTL mRNA-level and cannot directly demonstrate diagnostic performance of circulating serum FTL protein. To date, serum FTL research has predominantly focused on vascular diseases (20, 21), with few studies exploring its utility as a diagnostic or prognostic biomarker in cancers. As the functional subunit of ferritin, FTL exhibits more specific changes in expression that may carry greater diagnostic value. Our study further validated the potential of serum FTL as a reliable and independent diagnostic biomarker for ESCC using serum samples from two independent cohorts (CZPH and SYSUCC). The reported sensitivity and specificity values in **Table 2** were derived from the interpolated optimal cutoff maximizing Youden’s index in MedCalc. Observed sensitivity calculated from discrete sample counts is provided in **Supplementary Table S3** for cross-verification. Serum FTL achieved a higher AUC than conventional serum markers for esophageal cancer, including CEA and SCCA. In the overall population, FTL yielded an AUC of 0.75 (95% CI: 0.674–0.816), while in early-stage ESCC it achieved an AUC of 0.838 (95% CI: 0.751–0.905) with a sensitivity of 86.36% and specificity of 77.92%. Notably, the diagnostic performance of these traditional markers in our study was consistent with previous reports in the literature (22). We further constructed a combined model incorporating FTL and SCCA, which resulted in a significantly increased AUC in the overall population (0.816 vs. 0.75, p = 0.0106). However, this combined model did not show a clear additional benefit in early-stage patients (AUC = 0.824 vs. 0.838, *P* = 0.6694), which may be partly explained by the relatively small sample size of early-stage cases in the present study (n = 20). A relatively small early-stage subgroup may lead to limited statistical power and slightly wider confidence intervals for the diagnostic performance analysis of early tumors. Thus, the clinical value of FTL for early ESCC screening warrants further validation in larger early-stage patient cohorts.

This study also found that FTL is highly expressed in epithelial cells and macrophages, key players in ESCC initiation and TME modulation. High FTL levels were linked to increased resting dendritic cells and M2 macrophages, but fewer Tfh cells. Resting dendritic cells may weaken anti-tumor responses, while M2 macrophages promote ESCC progression by suppressing immunity and aiding tumor invasiveness (23–25). Conversely, T follicular helper (Tfh) cells are crucial for B cell activation and antibody secretion, and their reduced infiltration may weaken humoral immunity in ESCC patients (26, 27). Within the GSE53624 cohort, we observed group-level differences in M2-macrophage fractions between FTL-high and FTL-low subgroups by Wilcoxon rank-sum test; however, no significant linear Pearson correlation was detected when treating FTL as a continuous variable. This discrepancy may arise from the non-linear relationship between FTL mRNA and M2 macrophage abundance, as well as different statistical assumptions between dichotomized subgroup comparison and continuous correlation analysis. These observations are consistent with a previous report demonstrating that FTL can recruit macrophages to the TME, reduce ferroptosis sensitivity and promote tumor metastasis (28).

Our correlational analyses indicate that FTL may be associated with features of an immunosuppressive TME and may be involved in ferroptosis-related signaling in ESCC. High FTL expression correlates with increased ferroptosis pathway activity, particularly upregulating suppressor genes more than driver genes, which raises the hypothesis that FTL may suppress ferroptosis in ESCC cells. Since ferroptosis is linked to tumor progression and drug resistance (29), these correlational observations suggest that FTL could plausibly contribute to ESCC progression and metastasis. FTL is therefore considered a candidate molecule for further mechanistic and therapeutic investigation, rather than a validated therapeutic target.

By performing in silico knockout of FTL, our computational analyses predict a potential functional role for this iron-related molecule in esophageal squamous cell carcinoma. Our findings extend the conventional understanding of FTL beyond iron metabolism and suggest that FTL may act as a putative upstream modulator of inflammatory activation, oxidative stress, and transcriptional reprogramming in the ESCC microenvironment. Mechanistically, virtual ablation of FTL triggered a coordinated response dominated by pro-inflammatory signaling pathways, including IL-17, TNF, and NF-κB cascades. These pathways are central to chronic inflammation-driven carcinogenesis and are tightly linked to disease progression and therapeutic resistance in ESCC. The identification of core hub genes, including FOS, ATF3, NFKBIA, and CXCL8, further supports a model in which FTL serves as a functional node that orchestrates inflammatory and immune-related gene networks. Notably, our analysis also uncovered a mechanistic link between FTL and redox homeostasis through interactions with TFRC, TXNRD1, and NFE2L2. This connection places FTL at the intersection of iron metabolism, reactive oxygen species regulation, and inflammatory signaling—a tripartite axis increasingly recognized as a driver of epithelial malignancies. Collectively, these in silico results indicate FTL may be an important contributing factor in ESCC. However, our findings are based solely on in silico knockout; thus, further *in vitro* and *in vivo* studies are warranted to validate these observations and clarify the underlying mechanisms.

Despite the promising diagnostic performance of serum FTL observed in the present study, several limitations of this study should also be acknowledged. First, well-characterized and highly specific antibody–antigen pairs targeting FTL remain limited. Second, no standardized or clinically validated commercial assay kit is available for serum FTL detection; most current methods rely on research-grade reagents, leading to poor inter-laboratory comparability and limited reproducibility. Third, the predominant molecular form of FTL in human serum has not been fully clarified. Circulating FTL exists as a heterogeneous mixture of free light chains, ferritin heteropolymers, glycosylated isoforms, and iron-bound complexes, which may affect antibody recognition and quantitative accuracy. Fourth, circulating FTL can be influenced by multiple clinical confounders including iron metabolism status, systemic inflammation, hepatic function, age and sex. Although major inflammatory and malignant confounders were excluded through our strict enrollment criteria, iron-related biomarkers and CRP were not measured in the present cohort, and residual confounding effects cannot be completely ruled out. Further investigations are therefore warranted to develop robust immunoassays, characterize circulating FTL species, control for these clinical covariates, and validate its clinical utility in large multi-center cohorts.

## Conclusion

In summary, multi-omics analyses identified FTL as a reliable independent diagnostic biomarker for early-stage ESCC, with superior performance to conventional serum markers. Correlative multi-omics and computational analyses suggest that FTL may be involved in ESCC progression via potential crosstalk with the tumor microenvironment, ferroptosis and inflammatory signaling pathways. These mechanistic observations are derived solely from in silico predictions and transcriptome-level associations. FTL represents a candidate deserving further experimental functional validation before it can be considered a therapeutic target.

## Data Availability

The raw serum proteomic data from this study were submitted to [IPROX: PXD085956]. Additional supporting data can be obtained from the corresponding author on reasonable request.
