# Supplementary material for: Multi-omics integration identifies serum FTL as a novel diagnostic biomarker for esophageal squamous cell carcinoma
Source: Front Immunol. 2026 Oct 28;17:1935836. doi: 10.3389/fimmu.2026.1935836 (PMC13751605; doi:10.3389/fimmu.2026.1935836)
Supplement: Supplementary file 1 [file DataSheet1.pdf]

Table S1. Diagnostic Value of FTL in ESCC Based on the GEO Dataset

Dataset	AUC (95%CI)	Sensitivity	Specificity	PPV	NPV	PLR	NLR
GSE20347	0.947 (0.805-0.973)	87.28%	87.37%	87.36%	87.29%	6.911	0.146
GSE23400	0.872 (0.799-0.932)	86.89%	73.50%	76.63%	84.86%	3.279	0.178
GSE53624	0.970 (0.944-0.986)	95.69%	89.52%	90.13%	95.41%	9.135	0.048

Table S2. Clinical characteristics of patients with ESCC

	ESCC			
	Overall	CZPH	SYSUCC	p-value
		n = 12	n = 66	
	n = 78	(15%)	(85%)	
**T**				0.182
1	11 (14.10%)	0 (0.00%)	11 (16.67%)	
2	15 (19.23%)	2 (16.67%)	13 (19.70%)	
3	40 (51.28%)	6 (50.00%)	34 (51.52%)	
4	12 (15.38%)	4 (33.33%)	8 (12.12%)	
**N**				0.081
0	17 (21.79%)	5 (41.67%)	12 (18.18%)	
1	17 (21.79%)	2 (16.67%)	15 (22.73%)	
2	27 (34.62%)	5 (41.67%)	22 (33.33%)	
3	17 (21.79%)	0 (0.00%)	17 (25.76%)	
**M**				>0.999
0	63 (80.77%)	10 (83.33%)	53 (80.30%)	
1	15 (19.23%)	2 (16.67%)	13 (19.70%)	
**Stage**				0.556
1	8 (10.26%)	0 (0.00%)	8 (12.12%)	
2	12 (15.38%)	3 (25.00%)	9 (13.64%)	
3	27 (34.62%)	4 (33.33%)	23 (34.85%)	
4	31 (39.74%)	5 (41.67%)	26 (39.39%)	
**Location**				0.004

Lower segment	27 (34.62%)	0 (0.00%)	27 (40.91%)
Middle segment	28 (35.90%)	7 (58.33%)	21 (31.82%)
Middle-lower segment	10 (12.82%)	2 (16.67%)	8 (12.12%)
Upper segment	7 (8.97%)	0 (0.00%)	7 (10.61%)
Upper-middle segment	6 (7.69%)	3 (25.00%)	3 (4.55%)

Table S3. Observed versus interpolated sensitivity of diagnostic biomarkers in early-stage ESCC

Biomarker	Cut-off value	Total (n)	TP	FN	Sensitivity (%)*	Observed Sensitivity (%)
CEA	>1.4 ng/mL	20	16	4	86.36	80.00
SCCA	>1.4 ng/mL	20	10	10	59.09	50.00
FTL	>241.079 ng/mL	20	17	3	86.36	85.00
Panel 2	>0.5229	20	14	6	72.73	70.00

Sensitivity (*) corresponds to the interpolated theoretical value output by MedCalc software, calculated by maximizing Youden's index. Observed sensitivity was directly computed from raw true-positive (TP) and false-negative (FN) counts at sample level. Observed Sensitivity = TP/(TP+FN)
