# Supplementary material for: Multi-omics integration identifies serum FTL as a novel diagnostic biomarker for esophageal squamous cell carcinoma
Source: Front Immunol. 2026 Oct 28;17:1935836. doi: 10.3389/fimmu.2026.1935836 (PMC13751605; doi:10.3389/fimmu.2026.1935836)
Supplement: Supplementary file 2 [file DataSheet2.pdf]

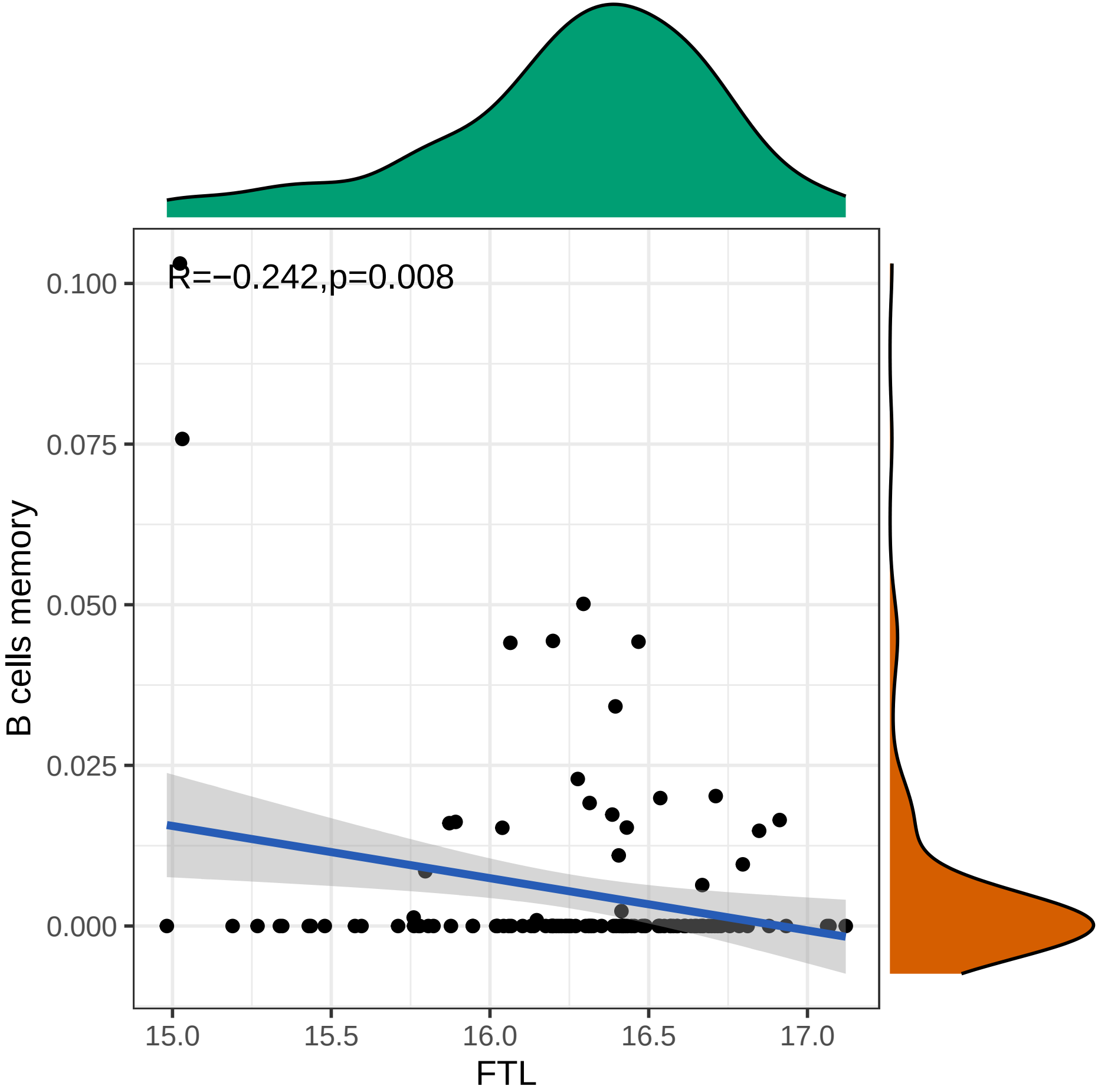
R=−0.242,p=0.008
B cells memory
0.100
0.075
0.050
0.025
0.000
15.0
15.5
16.0
16.5
17.0
FTL

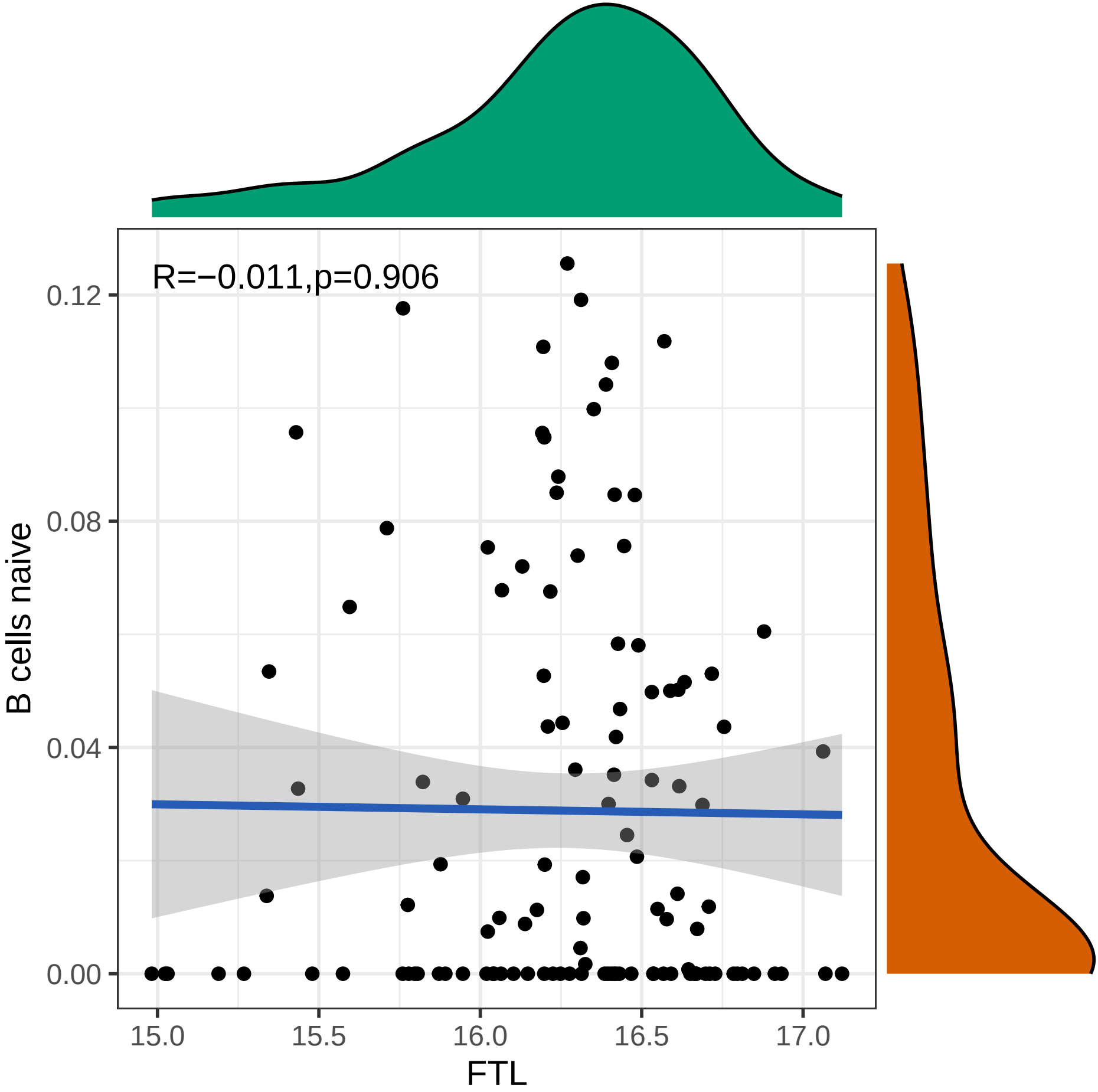

R=−0.011,p=0.906
0.12
0.08
0.04
0.00
B cells naive
15.0
15.5
16.0
16.5
17.0
FTL

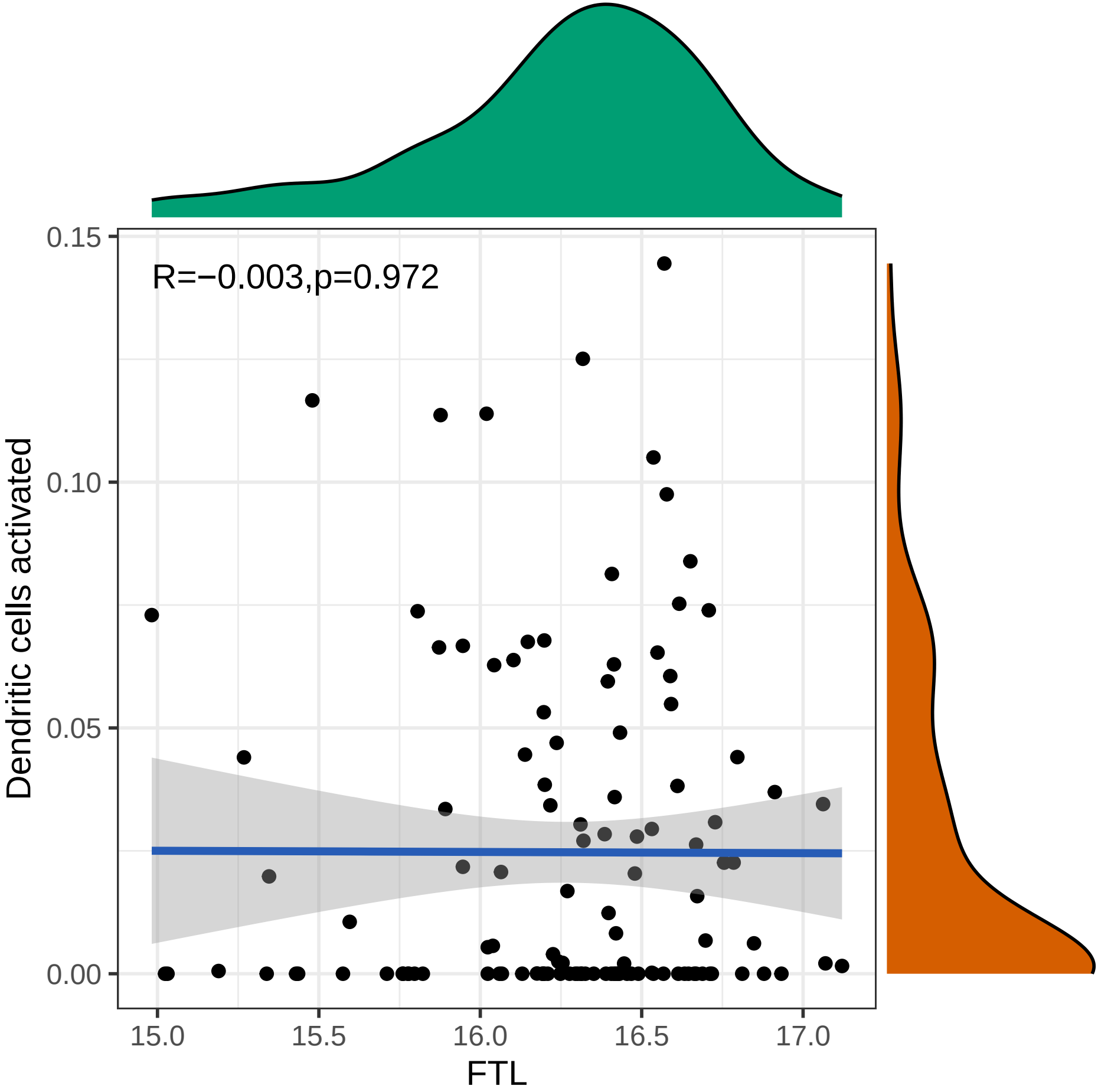

R=−0.003,p=0.972
0.15
0.10
0.05
0.00
Dendritic cells activated
15.0
15.5
16.0
16.5
17.0
FTL

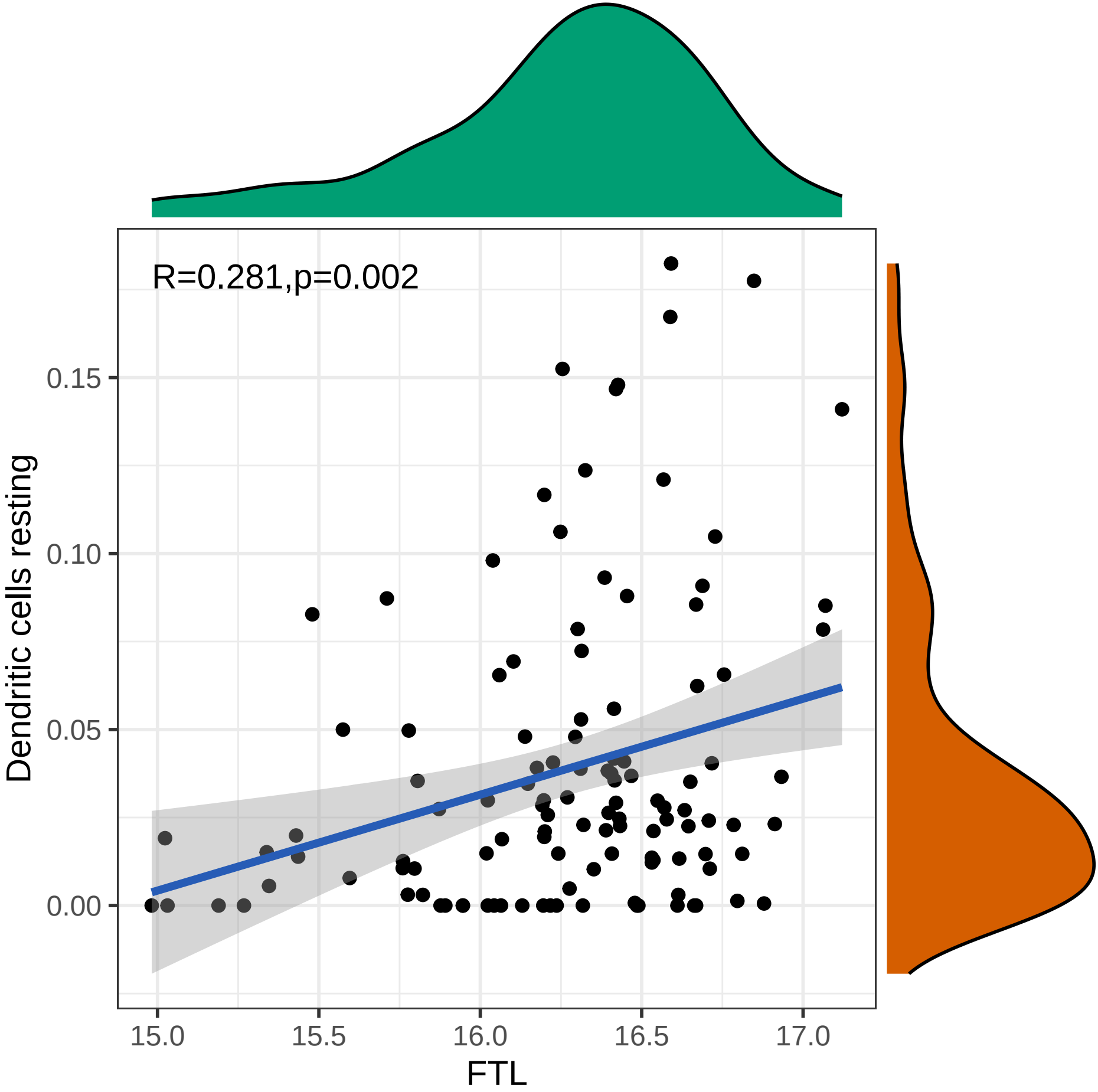

R=0.281,p=0.002
0.15
0.10
0.05
0.00
Dendritic cells resting
15.0
15.5
16.0
16.5
17.0
FTL

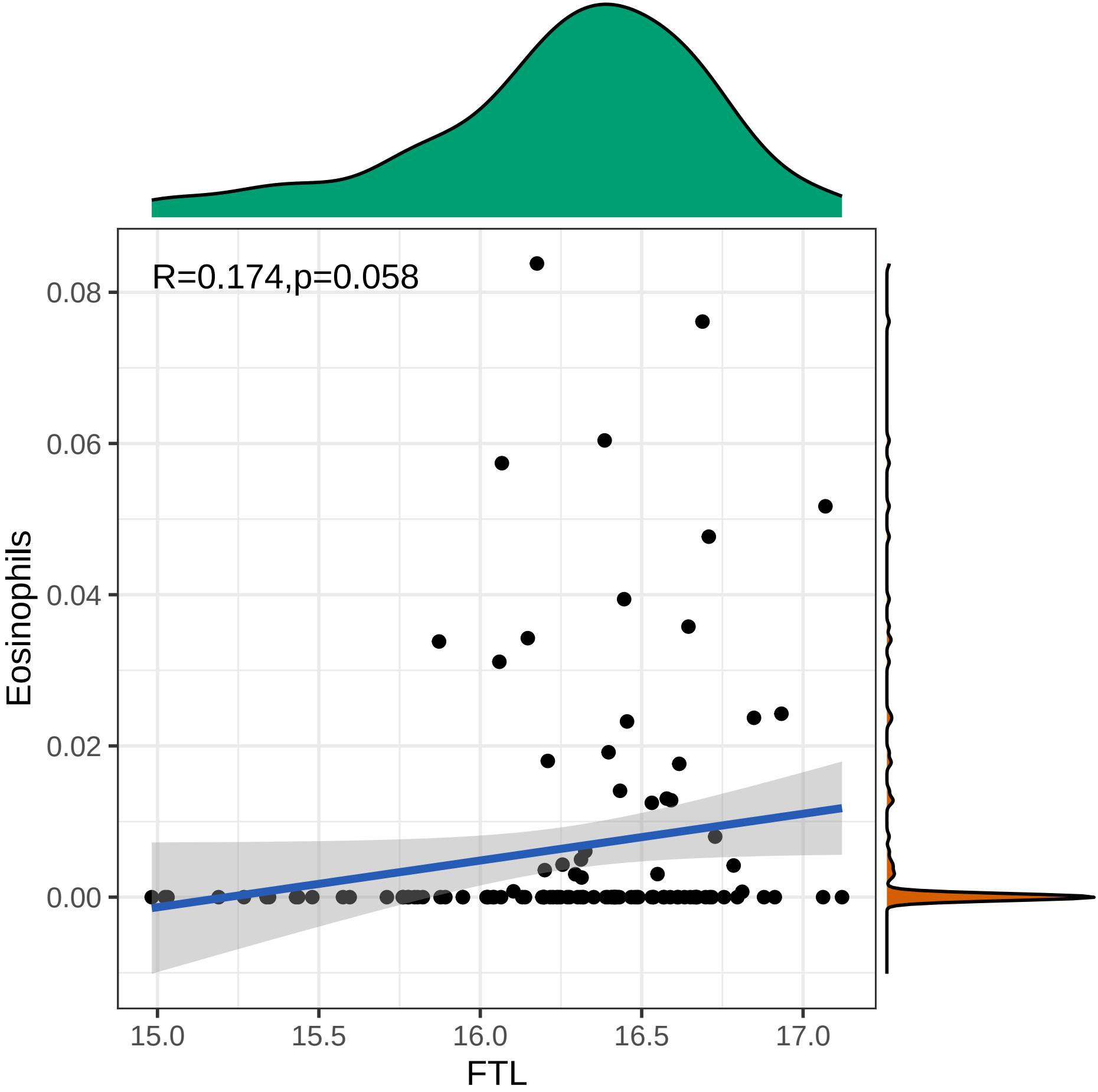
R=0.174,p=0.058
Eosinophils
0.08
0.06
0.04
0.02
0.00
15.0
15.5
16.0
16.5
17.0
FTL

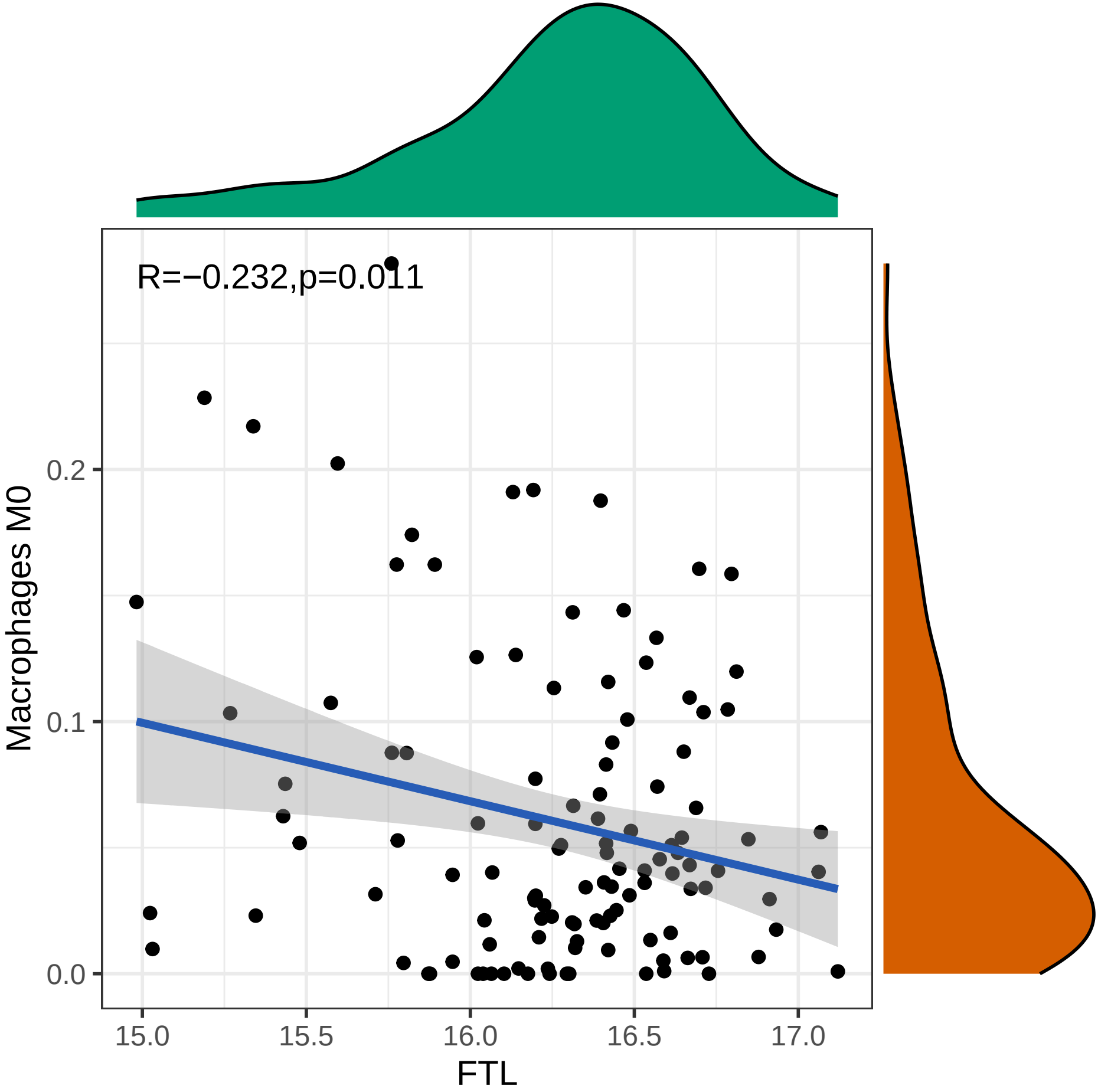

R=−0.232,p=0.011
Macrophages M0
0.0
0.1
0.2
15.0
15.5
16.0
16.5
17.0
FTL

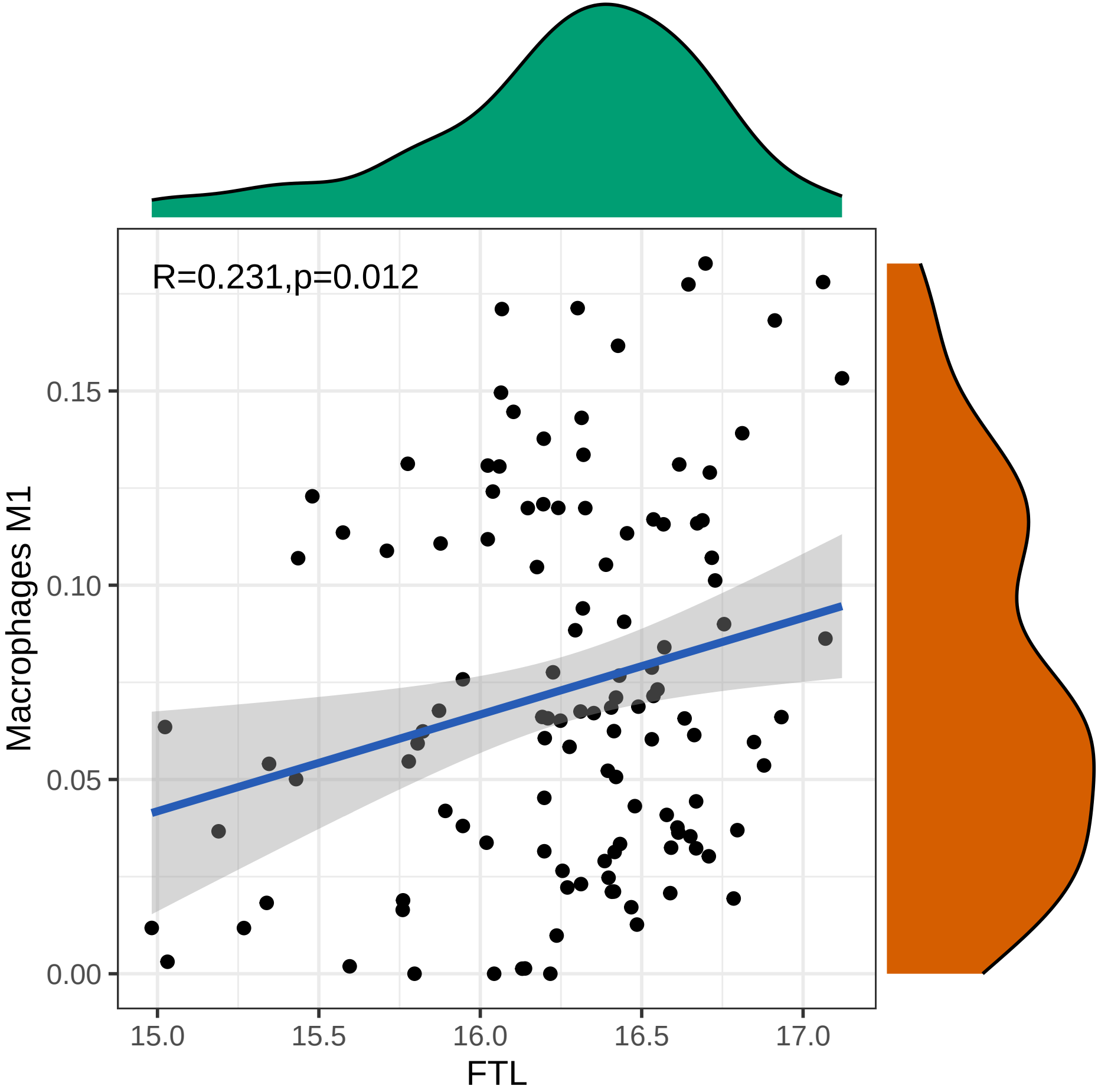
R=0.231,p=0.012
Macrophages M1
0.00
0.05
0.10
0.15
15.0
15.5
16.0
16.5
17.0
FTL

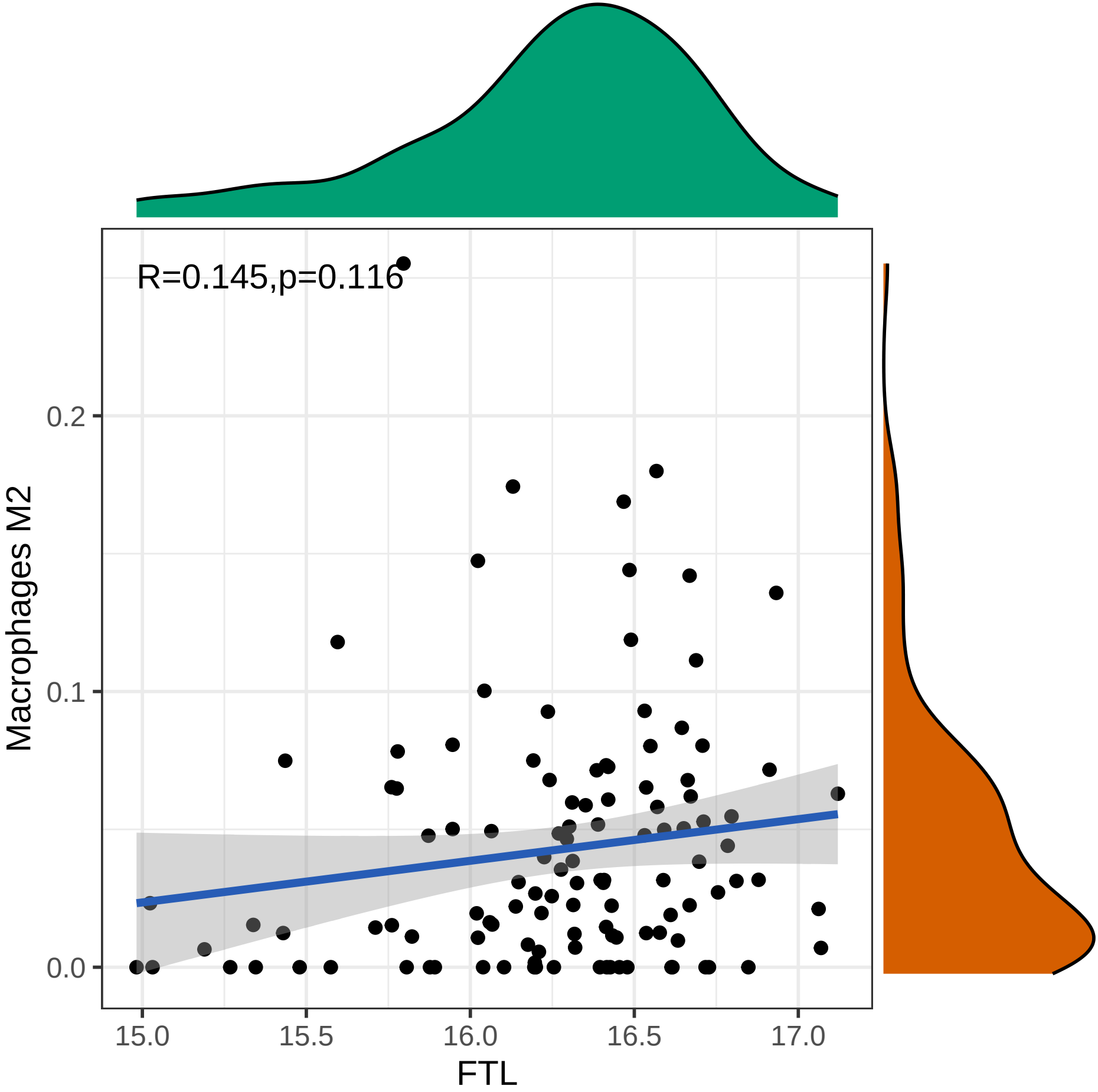

R=0.145,p=0.116
Macrophages M2
0.0
0.1
0.2
15.0
15.5
16.0
16.5
17.0
FTL

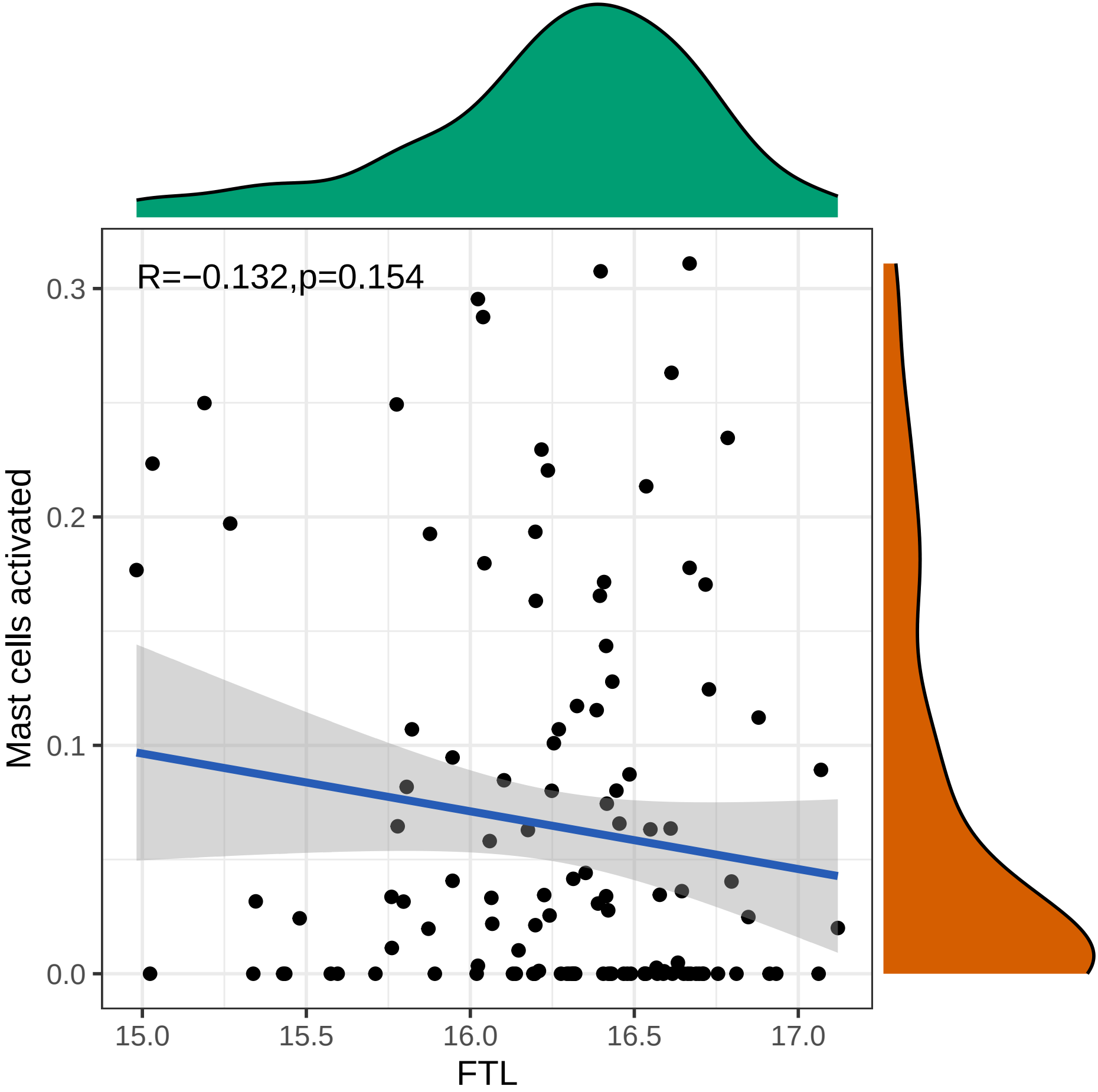
R=−0.132,p=0.154
0.3
0.2
0.1
0.0
Mast cells activated
15.0
15.5
16.0
16.5
17.0
FTL

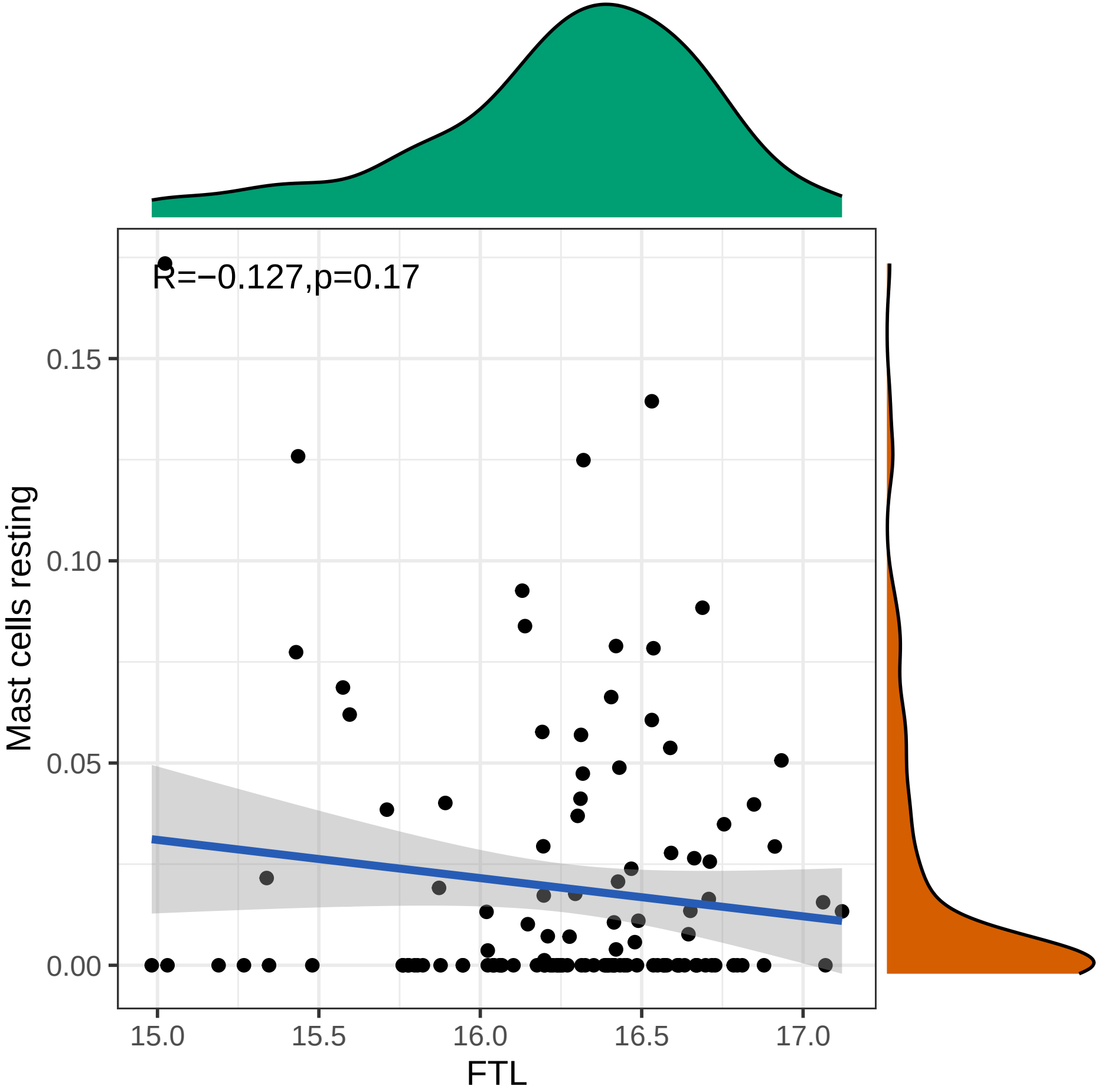

R=−0.127,p=0.17
Mast cells resting
0.15
0.10
0.05
0.00
15.0
15.5
16.0
16.5
17.0
FTL

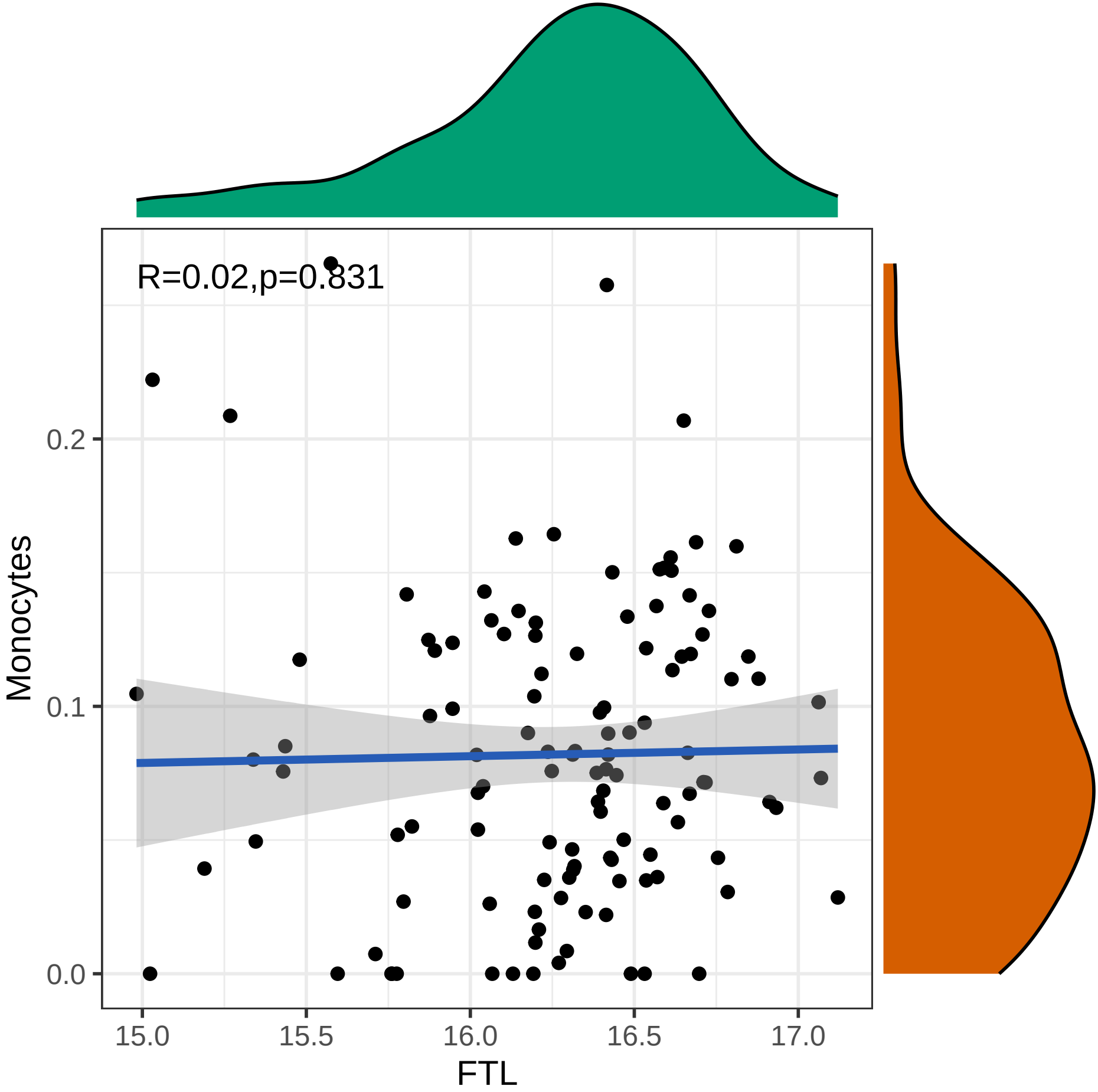

R=0.02,p=0.831
Monocytes
0.0
0.1
0.2
15.0
15.5
16.0
16.5
17.0
FTL

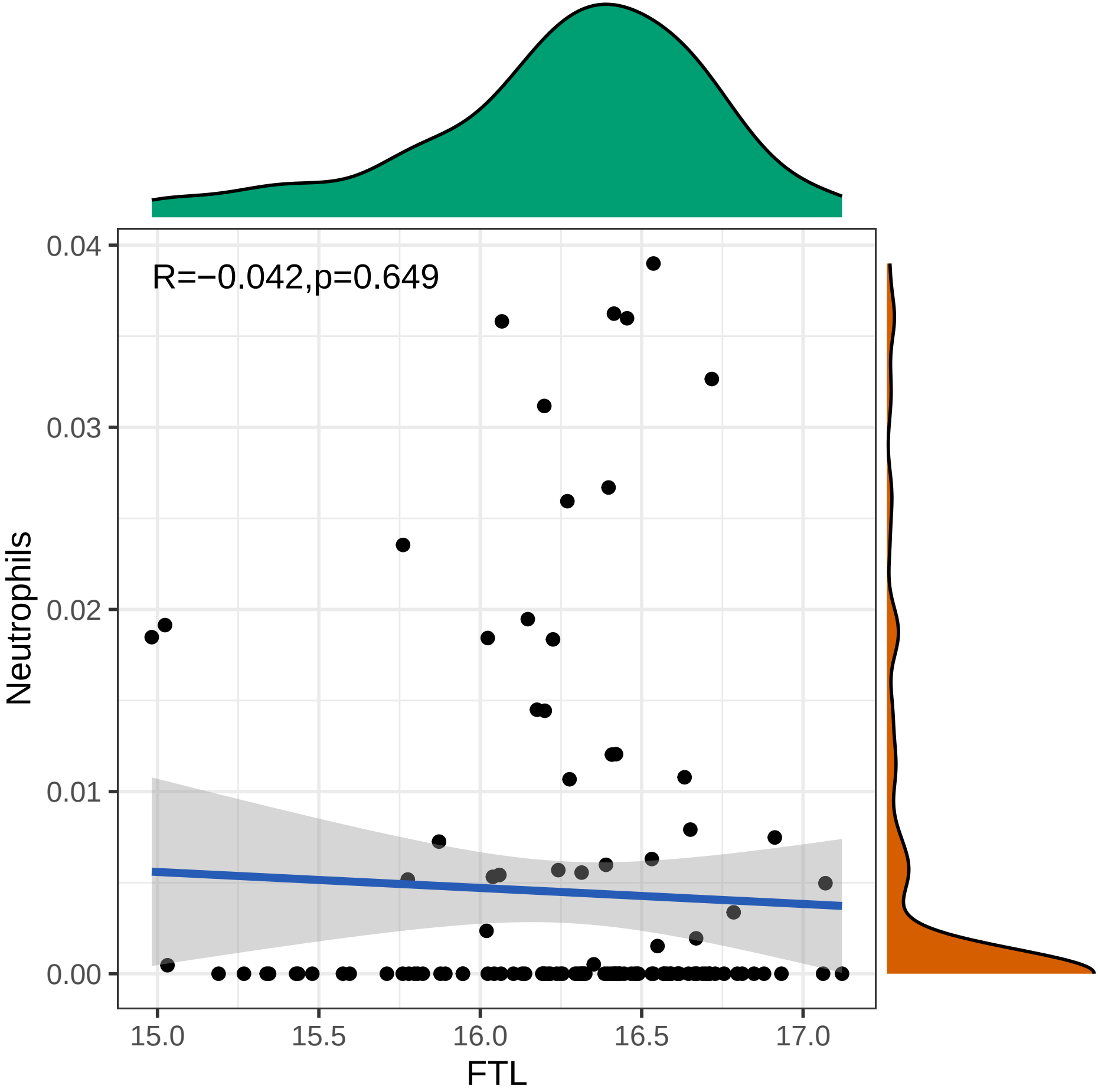
R=−0.042,p=0.649
0.04
0.03
0.02
0.01
0.00
Neutrophils
15.0
15.5
16.0
16.5
17.0
FTL

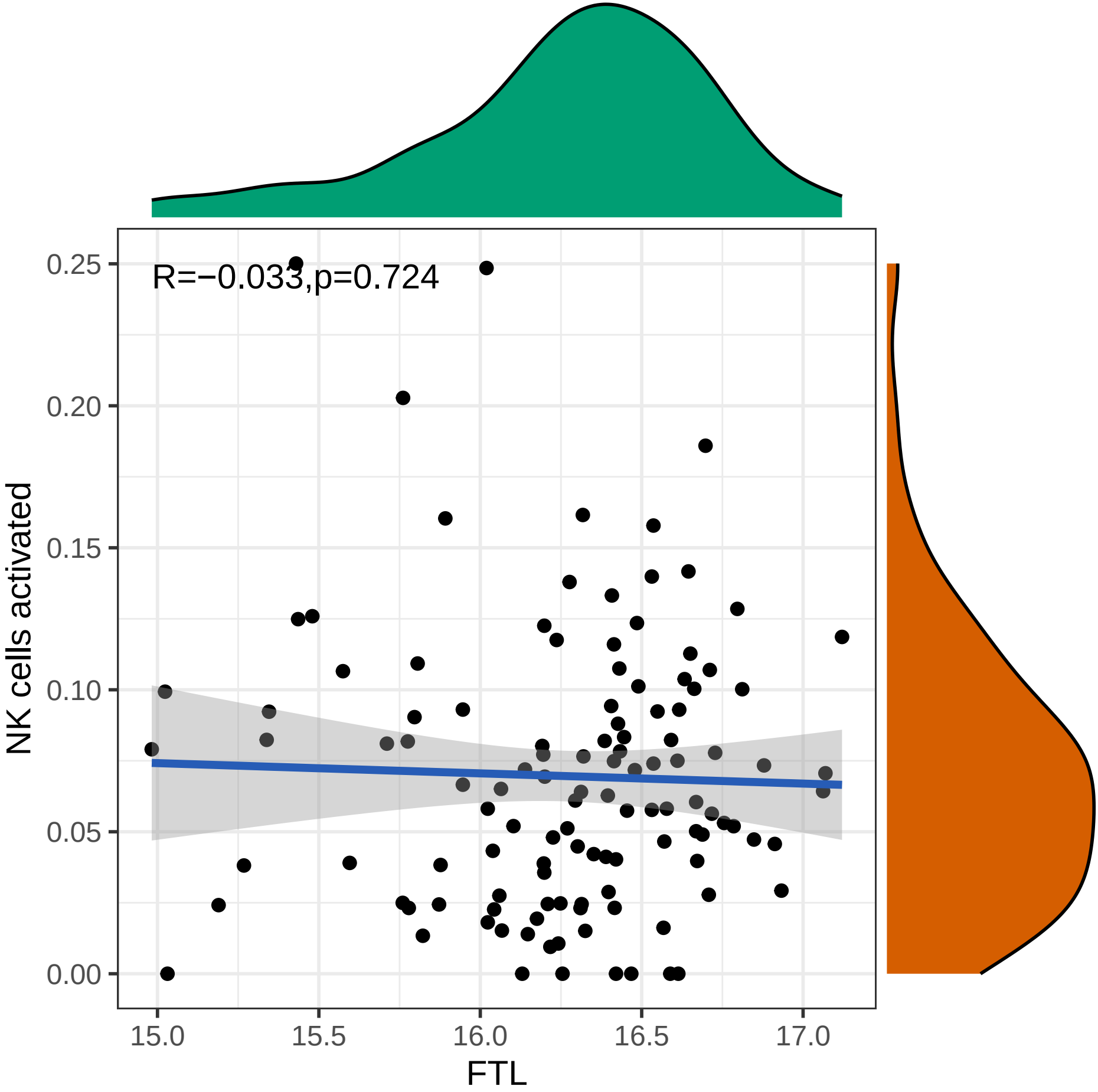

R=−0.033,p=0.724
NK cells activated
0.25
0.20
0.15
0.10
0.05
0.00
15.0
15.5
16.0
16.5
17.0
FTL

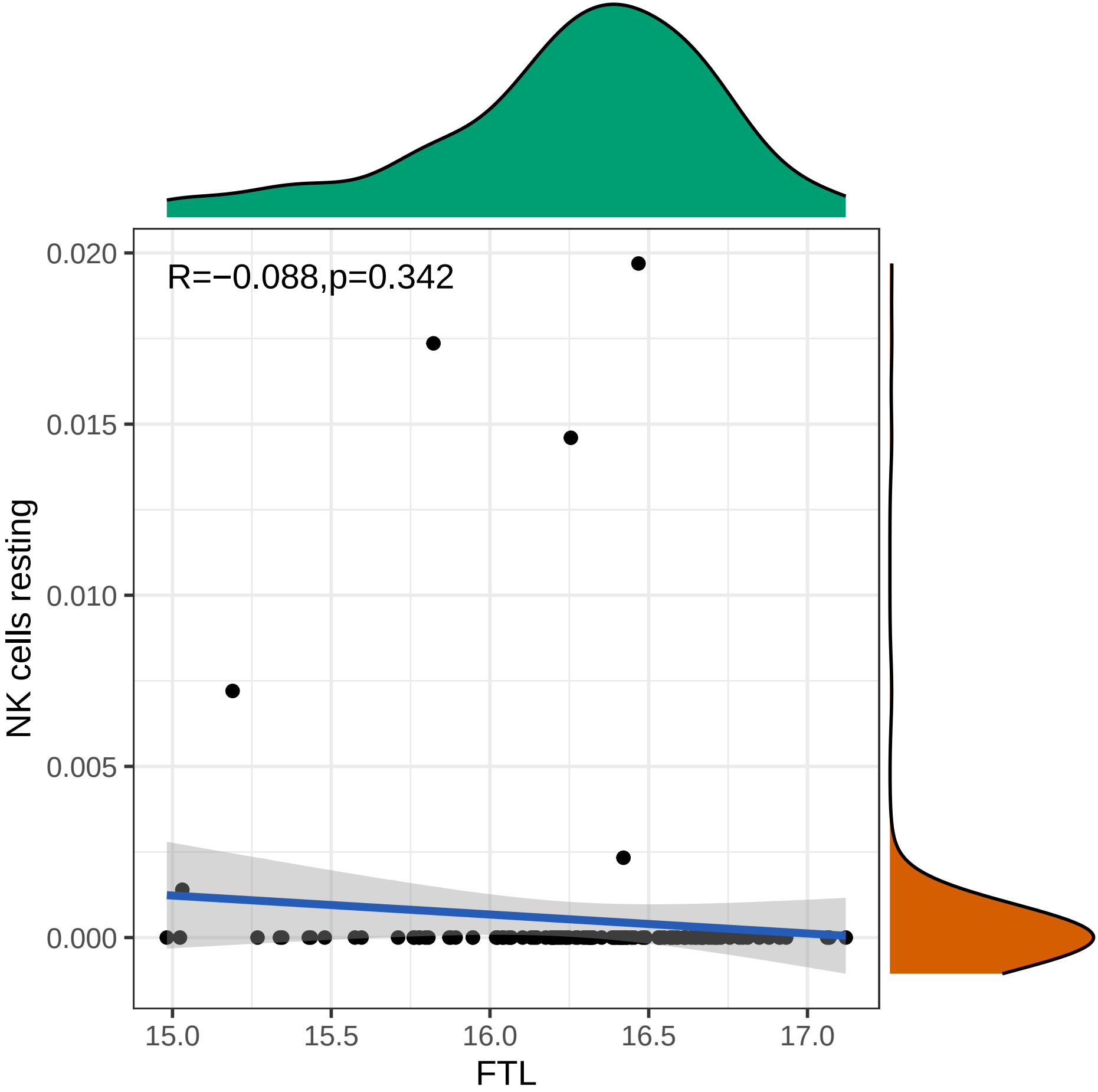

R=−0.088,p=0.342
0.020
0.015
0.010
0.005
0.000
NK cells resting
15.0
15.5
16.0
16.5
17.0
FTL

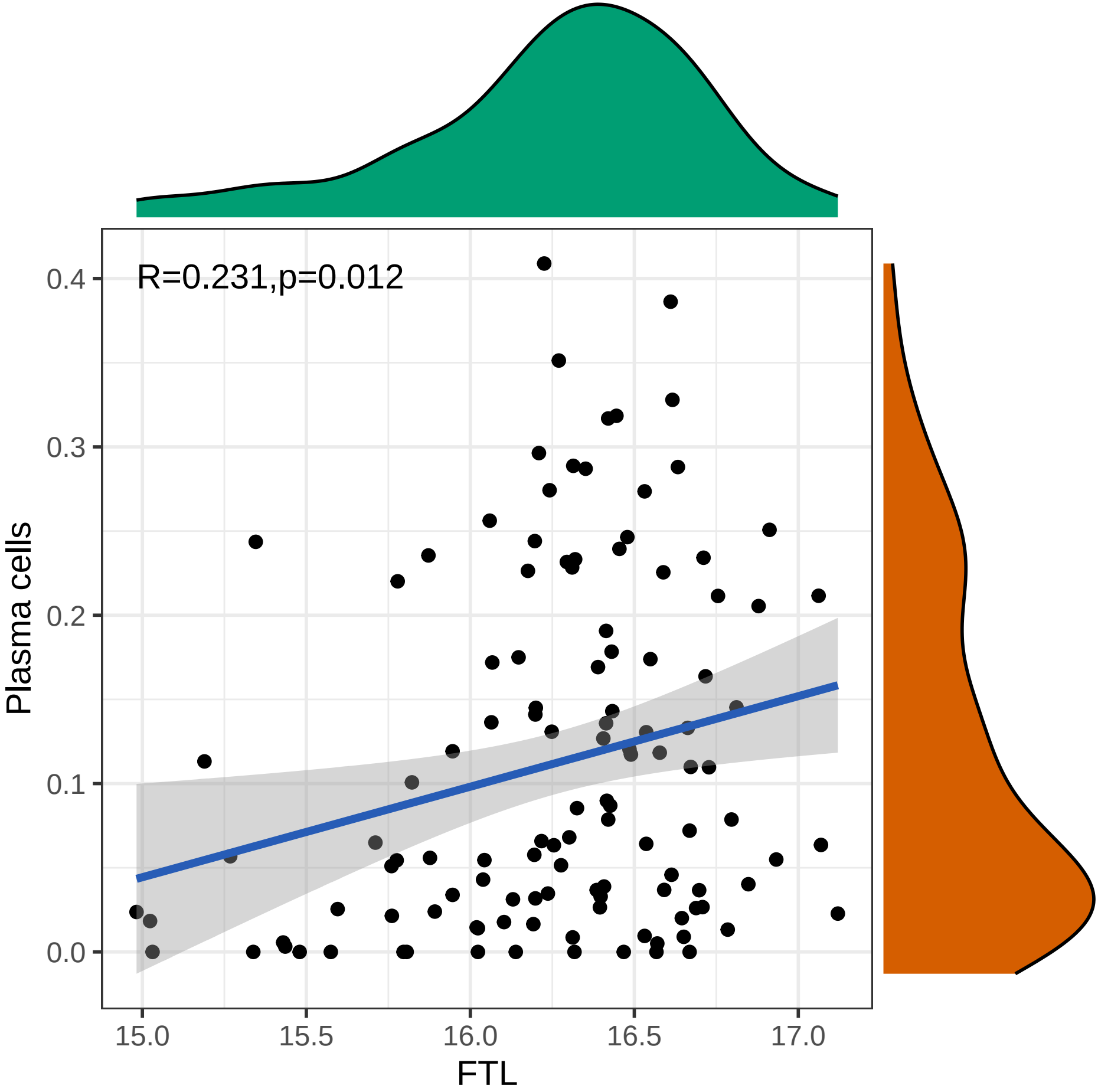
R=0.231,p=0.012
0.4
0.3
0.2
0.1
0.0
Plasma cells
15.0
15.5
16.0
16.5
17.0
FTL

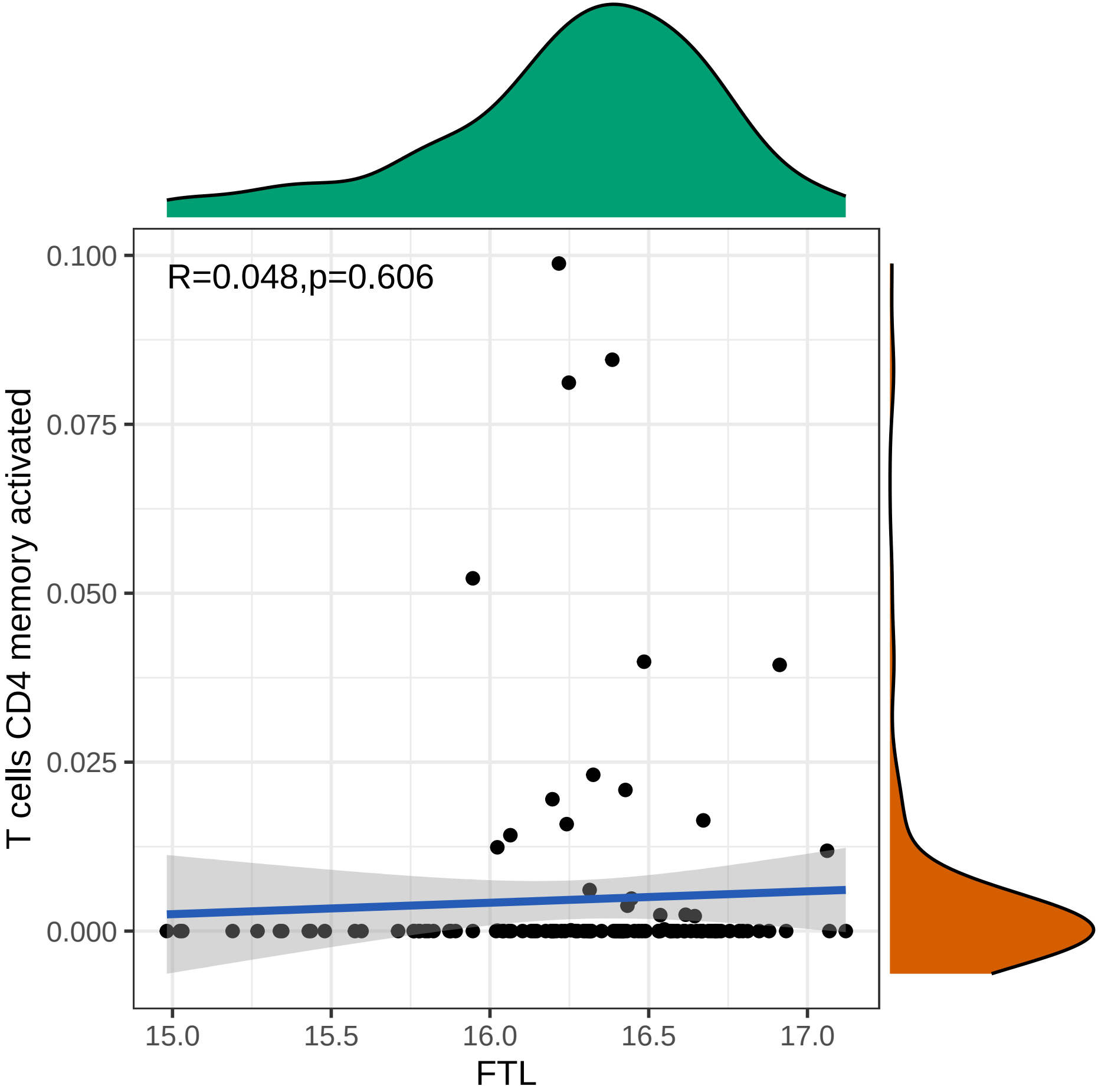

R=0.048,p=0.606
0.100
0.075
0.050
0.025
0.000
T cells CD4 memory activated
15.0
15.5
16.0
16.5
17.0
FTL

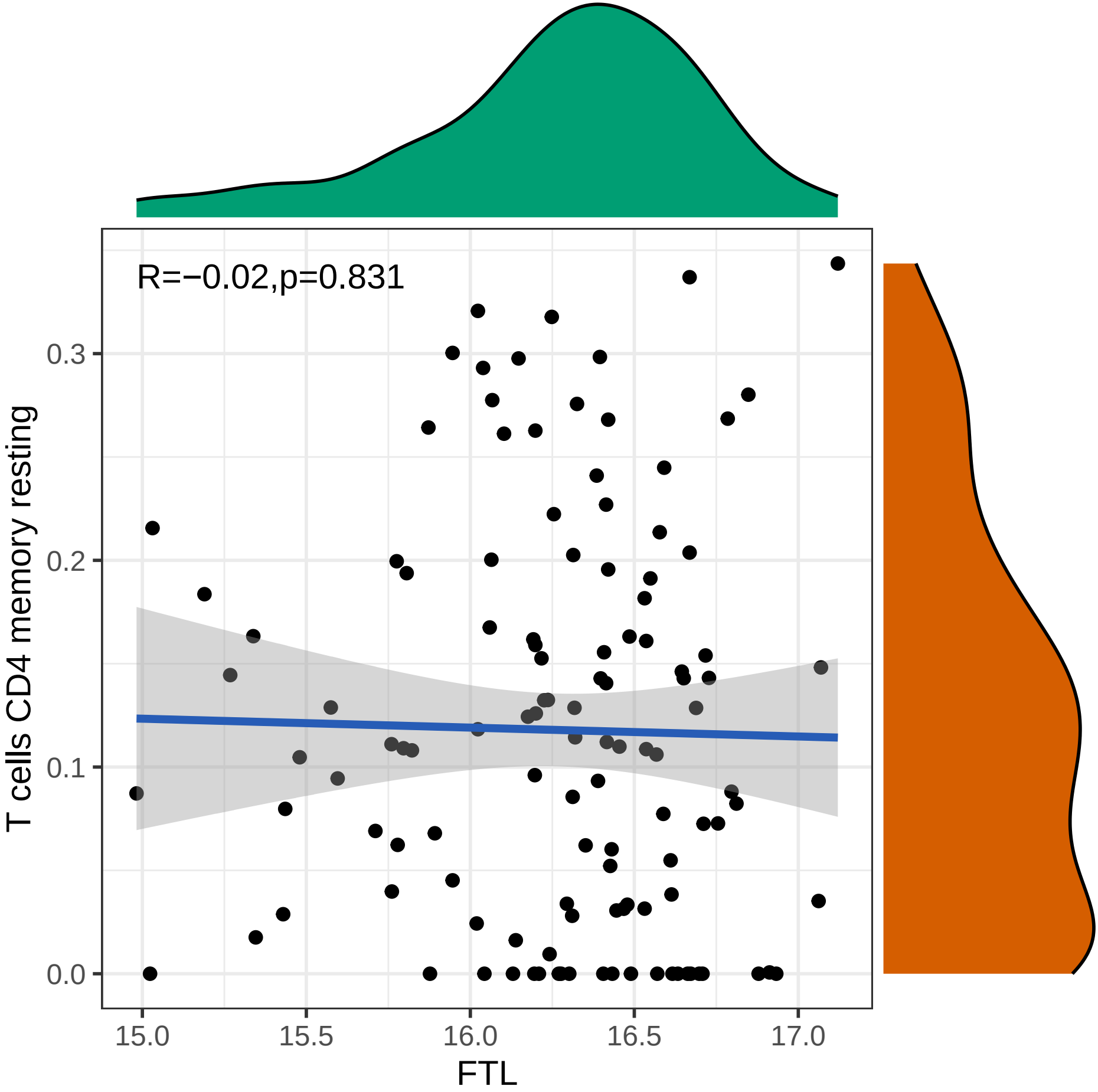
R=−0.02,p=0.831
0.3
0.2
0.1
0.0
T cells CD4 memory resting
15.0
15.5
16.0
16.5
17.0
FTL

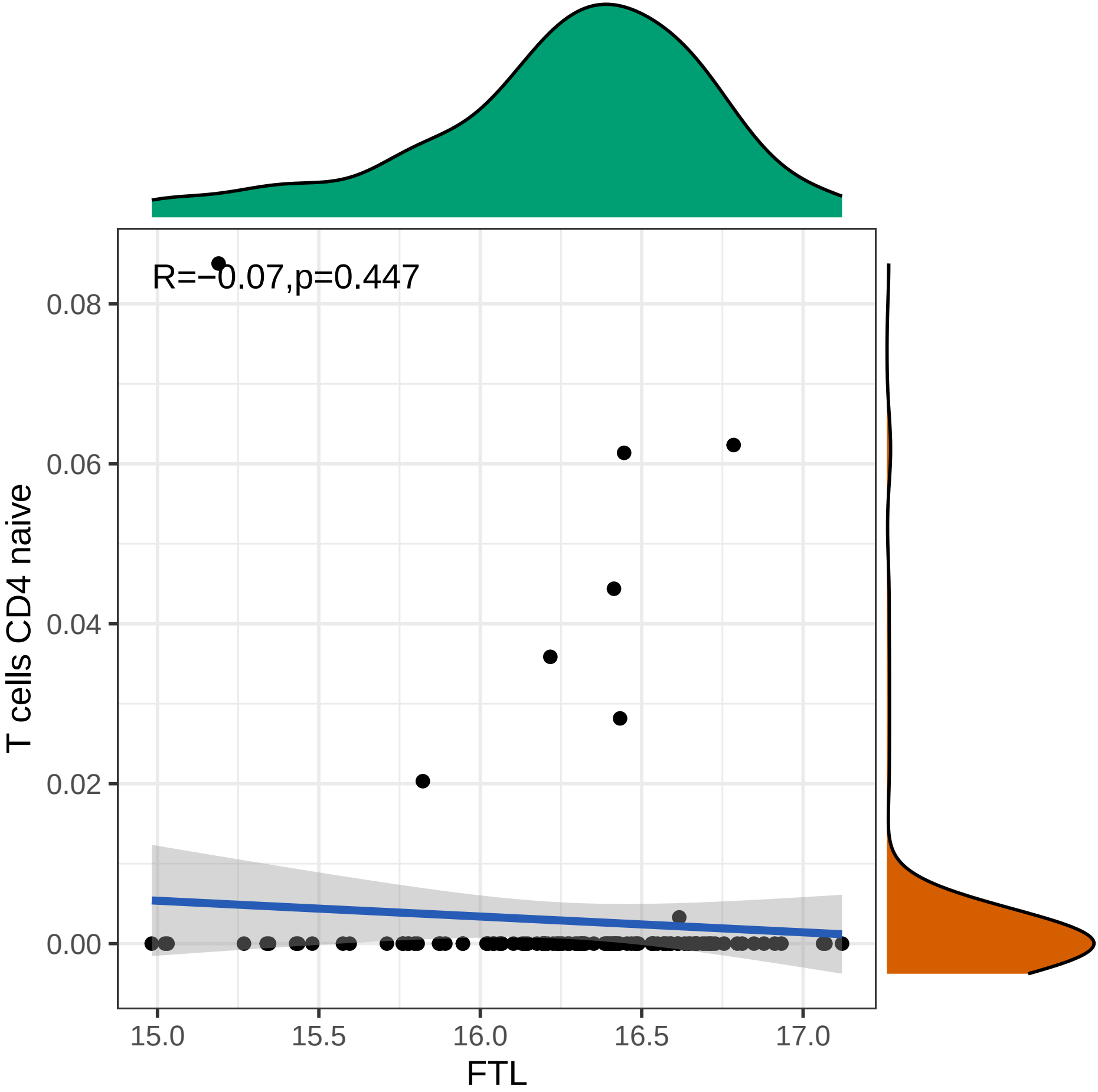
R=−0.07,p=0.447
0.08
0.06
0.04
0.02
0.00
T cells CD4 naive
15.0
15.5
16.0
16.5
17.0
FTL

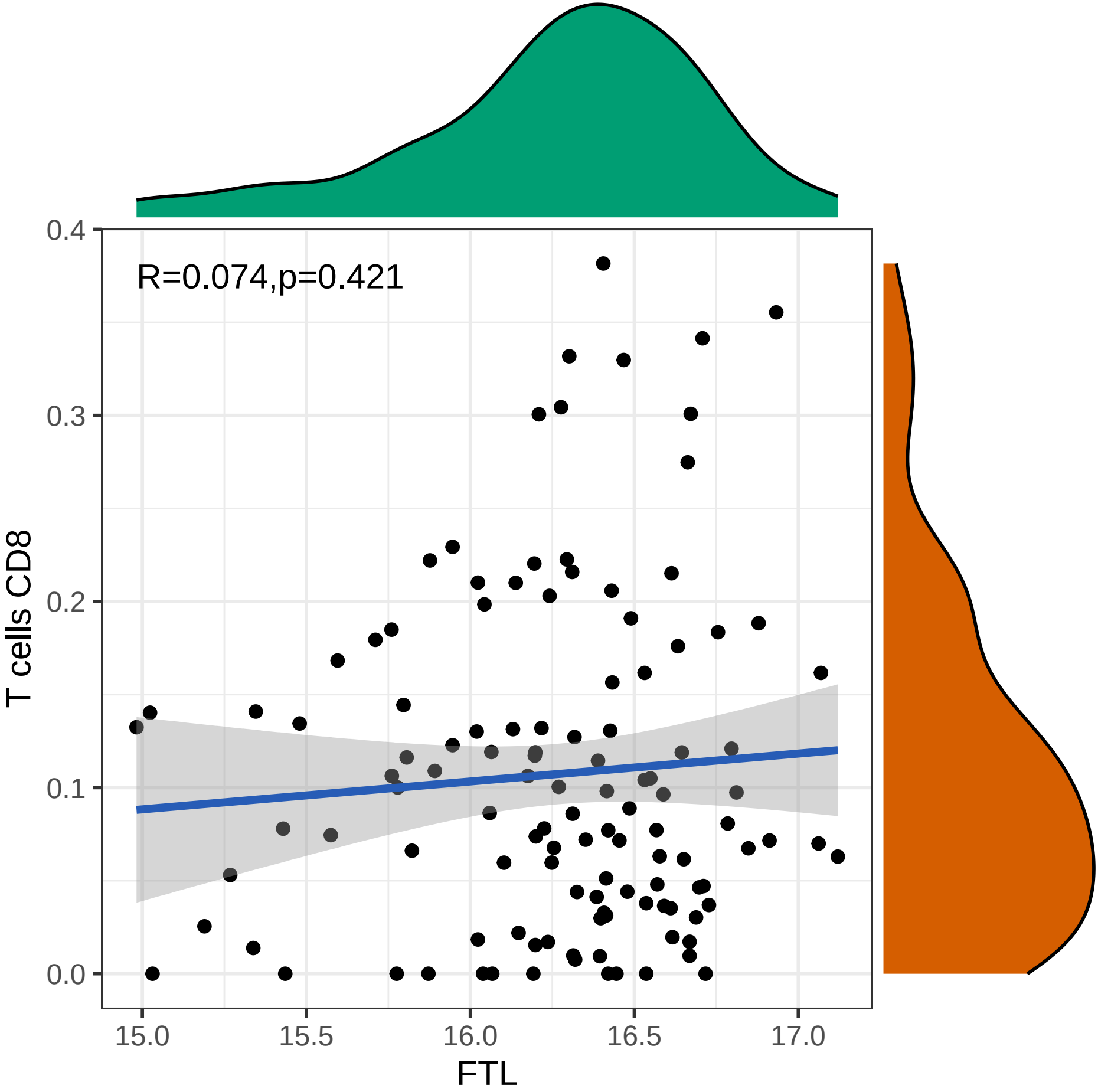

R=0.074,p=0.421
0.4
0.3
0.2
0.1
0.0
T cells CD8
15.0
15.5
16.0
16.5
17.0
FTL

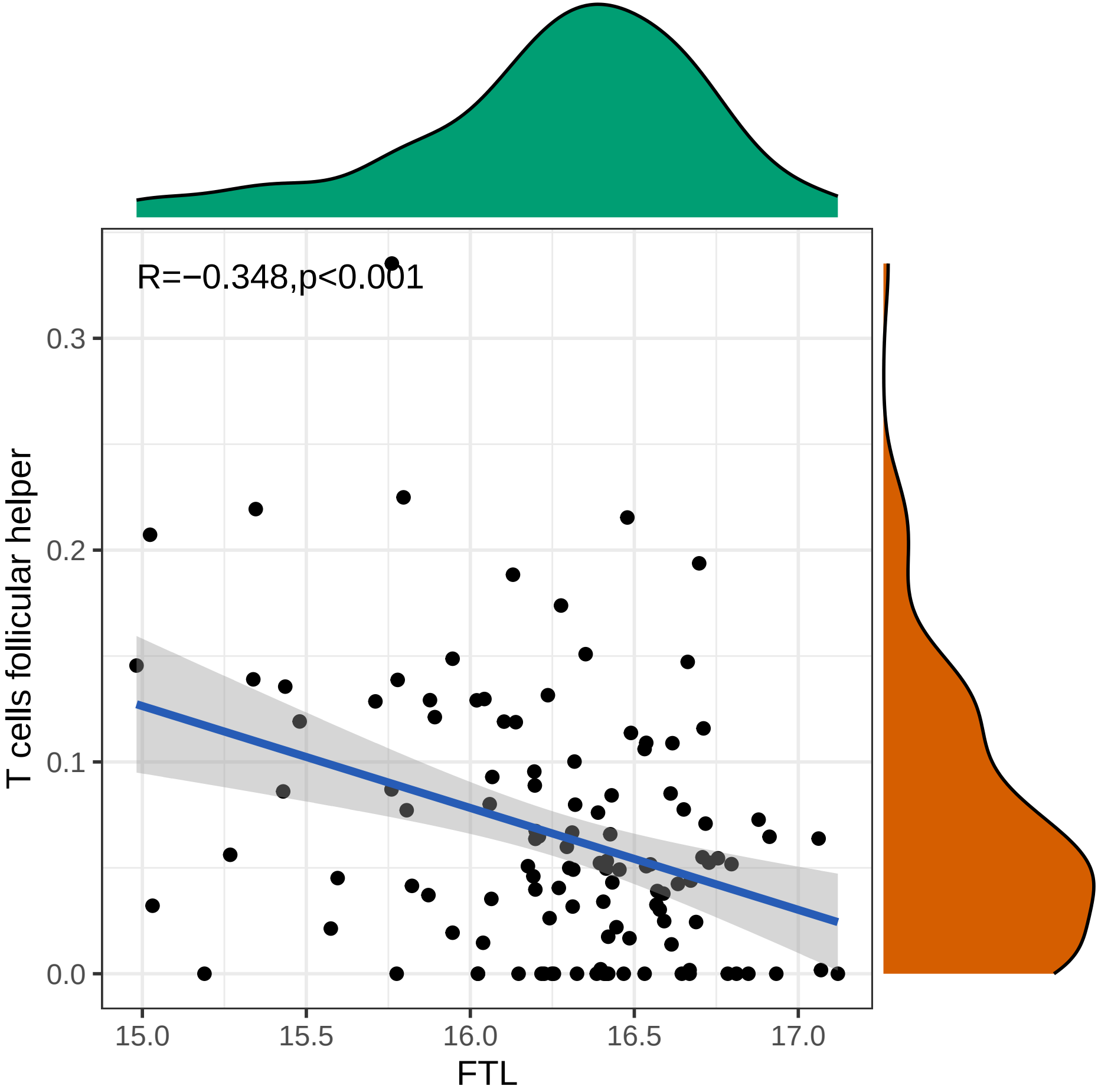

R=−0.348,p<0.001
0.3
0.2
0.1
0.0
T cells follicular helper
15.0
15.5
16.0
16.5
17.0
FTL

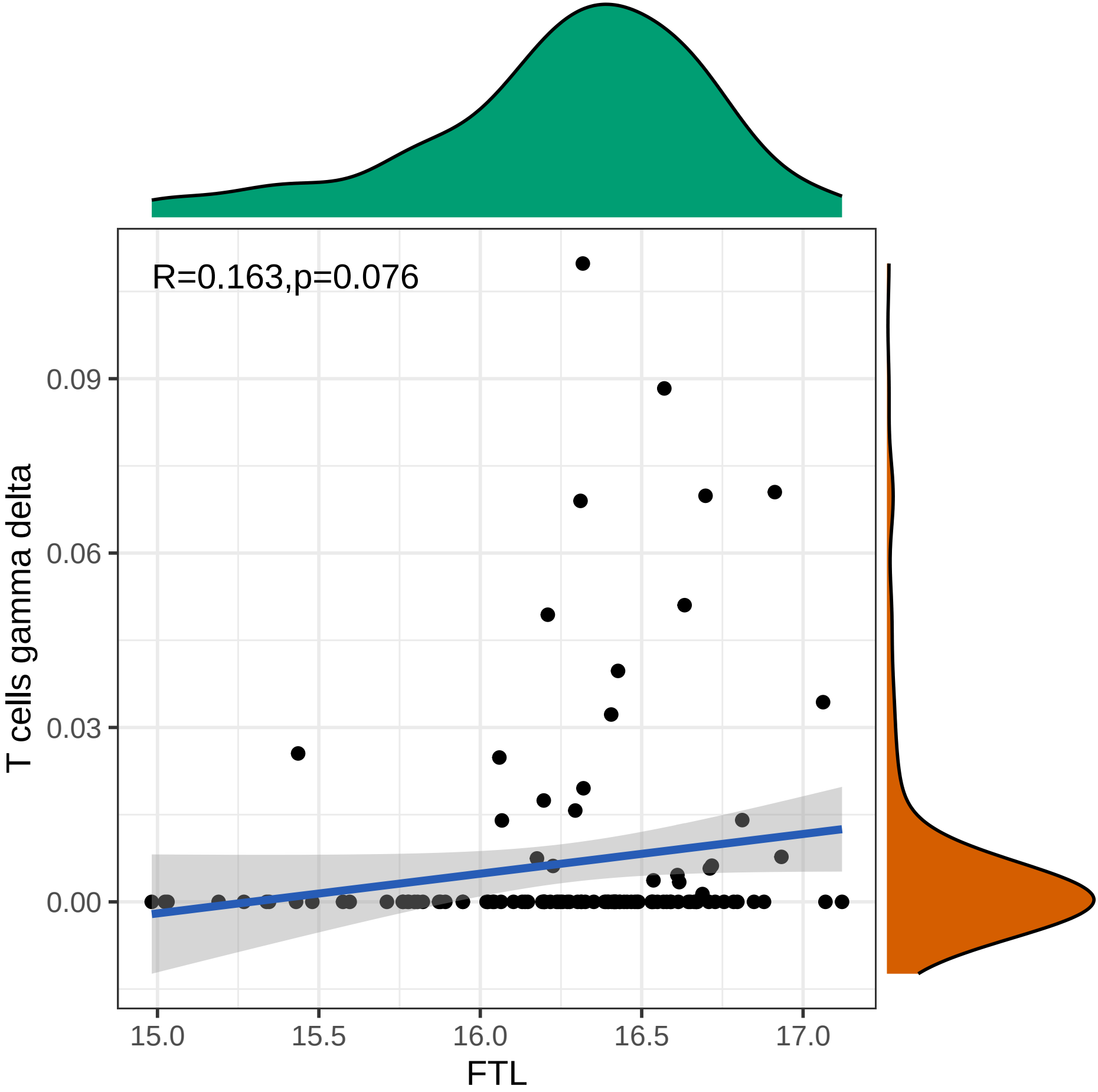

R=0.163,p=0.076
T cells gamma delta
0.09
0.06
0.03
0.00
15.0
15.5
16.0
16.5
17.0
FTL

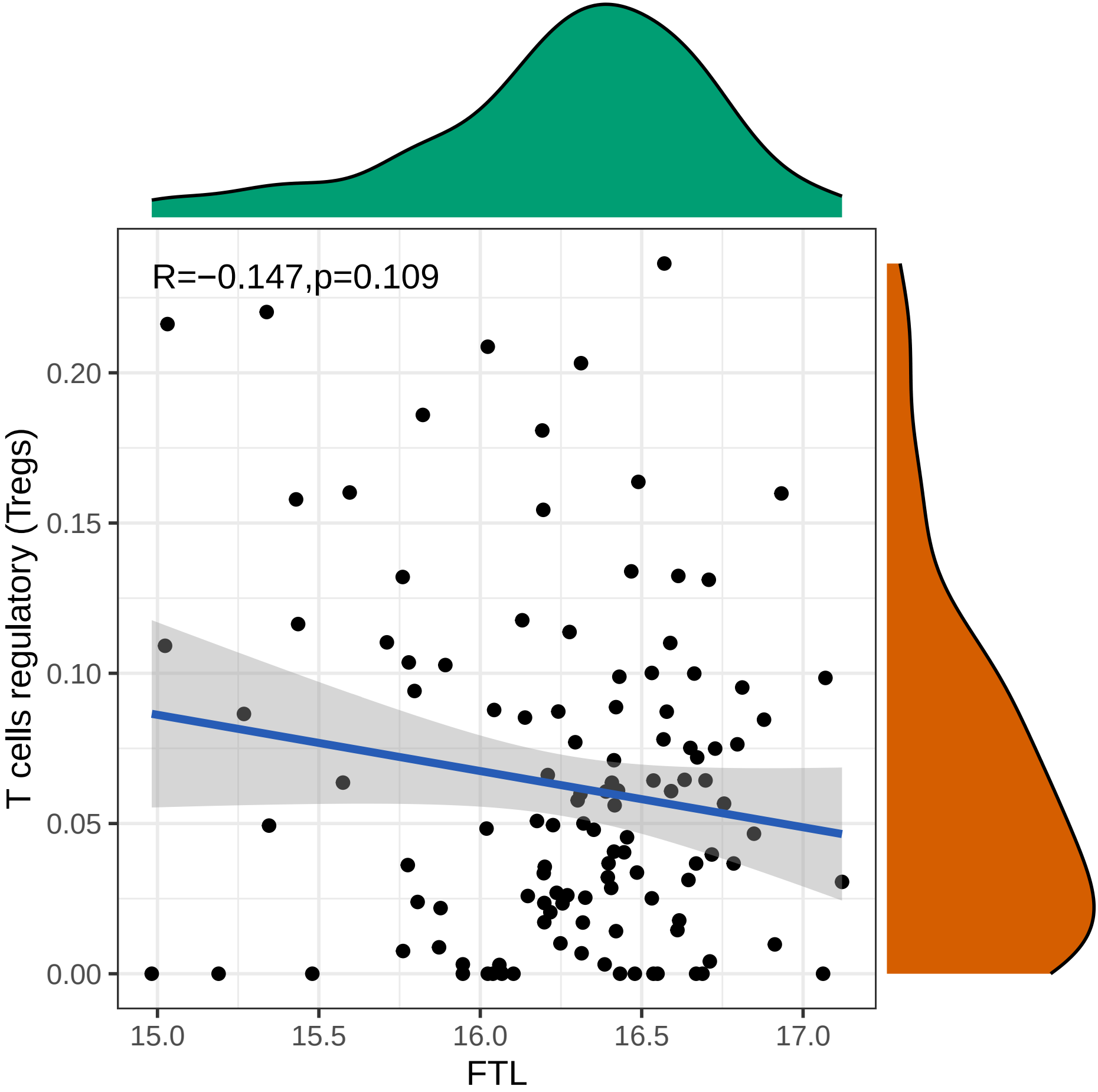

R=−0.147,p=0.109
T cells regulatory (Tregs)
0.00
0.05
0.10
0.15
0.20
15.0
15.5
16.0
16.5
17.0
FTL
